# Agarose gel microcapsules enable easy-to-prepare, picolitre-scale, single-cell genomics, yielding high-coverage genome sequences

**DOI:** 10.1038/s41598-022-20923-z

**Published:** 2022-10-18

**Authors:** Hiroyoshi Aoki, Yuki Masahiro, Michiru Shimizu, Yuichi Hongoh, Moriya Ohkuma, Yutaka Yamagata

**Affiliations:** 1 Ultrahigh Precision Optics Technology Team, Advanced Photonics Technology Group, RIKEN Center for Advanced Photonics, 3-1, Hirosawa, Wako, Saitama 351-0198 Japan; 2 Japan Collection of Microorganisms (JCM), RIKEN BioResource Research Center, 3-1-1, Koyadai, Tsukuba, Ibaraki 305-0074 Japan; 3 School of Life Science and Technology, Tokyo Institute of Technology, Tokyo, Japan

**Keywords:** Whole genome amplification, Symbiosis, Assay systems

## Abstract

A novel type of agarose gel microcapsule (AGM), consisting of an alginate picolitre sol core and an agarose gel shell, was developed to obtain high-quality, single-cell, amplified genomic DNA of bacteria. The AGM is easy to prepare in a stable emulsion with oil of water-equivalent density, which prevents AGM aggregation, with only standard laboratory equipment. Single cells from a pure culture of *Escherichia coli*, a mock community comprising 15 strains of human gut bacteria, and a termite gut bacterial community were encapsulated within AGMs, and their genomic DNA samples were prepared with massively parallel amplifications in a tube. The genome sequencing did not need second-round amplification and showed an average genome completeness that was much higher than that obtained using a conventional amplification method on the microlitre scale, regardless of the genomic guanine–cytosine content. Our novel method using AGM will allow many researchers to perform single-cell genomics easily and effectively, and can accelerate genomic analysis of yet-uncultured microorganisms.

## Introduction

Microorganisms are ubiquitously distributed in diverse environments. They are often associated with other organisms and play important roles in ecosystems. However, the majority of microbial species are difficult to culture with conventional methods and are called yet-uncultured microorganisms^1^. In the past two decades, they have been extensively studied using culture-independent methods such as amplicon sequencing analysis of small subunit ribosomal RNA (SSU rRNA) genes and shotgun sequencing analysis of metagenomes. Metagenomics is a powerful tool for investigating the ecological and physiological functions of microbiota based on their encoded genetic repertoires. The recent development of the computational binning of metagenomic fragments into respective microbial taxonomic assemblies has reinforced the utility of metagenomics^2,3^. However, computational binning based on the sequence composition, homology to database sequences, and sequence read coverage of each fragment can often fail to discriminate genomes of closely related (sub)species and to correctly sort genomic regions exhibiting distinct features from others, such as rRNA genes, prophages and plasmids^4,5^. Furthermore, a massive sequencing effort is required to obtain the genome sequences of minor species in the microbial community^3^.

Single-cell genomics, in which whole genomic DNA is amplified from physically isolated single cells^6^, has been used as an alternative or complementary method to reveal the metabolic capacity of yet-uncultured microorganisms^1,7^. Single-cell isolation can be achieved in many cases by using technologies such as fluorescence-activated cell sorting (FACS)^1^, micromanipulators^8^, microfluidic devices^9,10^ and encapsulation in water-in-oil droplets^11^. Although various methods for whole genome amplification have been developed^12^, multiple displacement amplification (MDA) using phi29 DNA polymerase with random primers is most widely used due to its relative simplicity, reliability and applicability^6,13^. However, MDA inherently demonstrates extreme amplification bias among genome regions^1^, which has been the major technical limitation in single-cell genomics. Additionally, the high guanine–cytosine (GC) content of genomic DNA tends to increase the amplification bias^1,14^. Amplification bias can be suppressed using small reaction vessels; for example, microchannel chambers (60 nL)^10^, nanolitre microwells (12 nL)^15^, virtual microfluidics (MDA in a gel sheet, 60 nL)^16^, digital droplets generated by using a microchannel (9.5–240 pL)^17–19^ and gel beads^20^. However, these microfabrication techniques are not available for many microbiologists, and the amount of the amplification product is often too little for direct genome sequencing; therefore, a second round of MDA is often required, which ultimately enhances the amplification bias^21^.

A hollow-core hydrogel microcapsule^22^, which consists of a hydrogel shell and a sol core, has potential as an efficient vessel for single-cell MDA. Many hollow-core hydrogel microcapsules have been reported to be useful for cell cultivation and cell transplantation^23–25^. However, the MDA product from single-cell genomic DNA amplified in a picolitre-scale hollow core in a microcapsule has not been fully evaluated for single-cell genomics.

**Figure 1:**
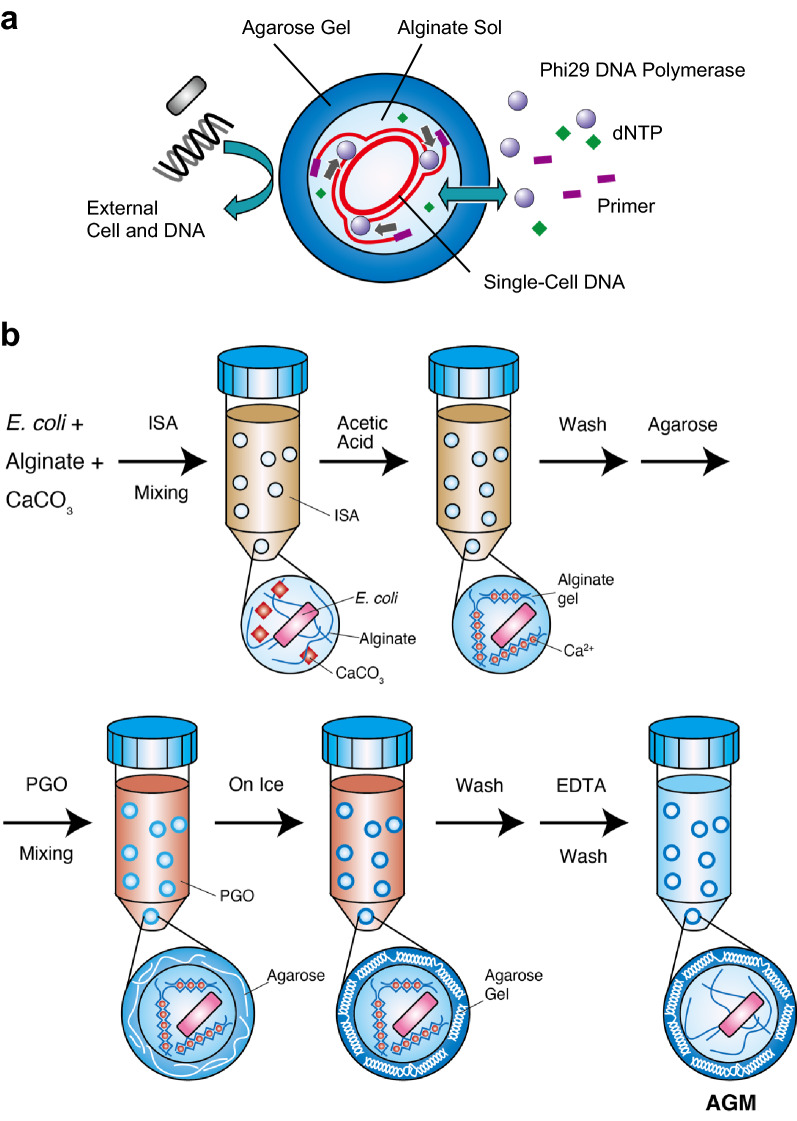
Schematic view of an agarose gel microcapsule (AGM) and its preparation process. (**a**) Schematic diagram of the AGM developed in this study for single-cell isolation and multiple displacement amplification (MDA). The AGM consists of an agarose hydrogel shell and an alginate sol core. The alginate core provides a picolitre-scale reaction chamber for MDA, while the agarose shell acts both as a permeable envelope for enzymes and small molecules and as a protective wall against larger particles and molecules such as bacterial cells and genomic/amplified DNA. (**b**) Preparation scheme for AGMs containing *Escherichia coli* cell(s). The alginate cores and agarose shells are gelated with Ca^2+^ from CaCO_3_ and cooled in an emulsion, respectively. Polyglyceryl-6 octacaprylate (PGO) can suppress the aggregation of AGMs during agarose gelation by cooling. Finally, the alginate gel cores are solated with EDTA by chelating Ca^2+^. *ISA* isostearyl alcohol.

Here, we introduce a novel single-cell genomics method using an AGM consisting of an agarose gel shell and an alginate sol hollow core, which enables single-cell isolation and picolitre-scale MDA (Fig. 1a). The AGM fabrication was optimised here for easier preparation using inexpensive equipment and reagents, such as a vortex mixer, centrifuges, and commercially available reagents (Fig. 1b), although the size uniformity of AGM is sacrificed compared to the methods using microfabrication techniques^26,27^. Thus, this AGM can be easily prepared and is scalable for many microbiologists. By using AGM, a single bacterial cell can be encapsulated in its picolitre-scale sol core and then subjected to MDA just by exchanging solutions (designated here ‘MDA-in-AGM’). We demonstrate, using *E. coli*, a mock mixture of human gut bacteria, and the termite gut microbiota, that MDA-in-AGM enables massively parallel single-cell genomics with much improved genome completeness compared to a conventional method using FACS and microlitre-scale MDA (FACS-MDA).

## Results

### Preparation of AGMs containing single bacterial cells

For AGM cores, alginate gel beads containing bacterial cells were prepared using the emulsification/internal gelation method^27^ (Fig. 1b). Briefly, a mixture of alginate solution, bacterial cells and CaCO_3_ nanoparticles was emulsified with isostearyl alcohol (ISA). The resulting microdroplets were gelated with calcium ions that were released from CaCO_3_ nanoparticles by the addition of acetic acid to the water-in-oil emulsion. The concentration of bacterial cells was optimised using serial dilutions so that one alginate microdroplet contained one or no bacterial cells. The resulting alginate gel cores were washed sequentially with diethyl ether, 1-butanol and Tris–HCl (pH 7.5) buffer.

Agarose solution (SeaPlaque, Lonza; final concentration 2%) was then added to the alginate gel cores suspended in Tris–HCl (pH 7.5) at 35 °C, and the mixture was emulsified with 0.25% (v/v) Span 85 in polyglyceryl-6 octacaprylate (PGO) at 35 °C (Fig. 1b). PGO was used here because its density is water-equivalent (0.997 g/mL), and hence PGO stably emulsified water (Supplementary Fig. S1a), prevented agarose gel aggregation (S1b), and increased the diameters (S1c and S1d) and yields (S1e) of agarose gel droplets. The agarose droplets were gradually gelated in the stable emulsion by cooling at 4 °C. After PGO was removed by mixing with diethyl ether and further with 1-butanol, the alginate gel cores were solated with a one-tenth volume of 0.5 M ethylenediaminetetraacetic acid (EDTA). The solation of alginate cores was verified by labelling the alginate cores with rhodamine 123 and observing the diffusion of the dye into buffer when melting the agarose gel shells under the presence of EDTA whereas the alginate cores remained in a gel form with 50 mM CaCl_2_ (Supplementary Fig. S2). The alginate sol core provides an appropriate space for the MDA reaction, which was shown by the much greater yields of amplified DNA than in reactions with an alginate gel core or agarose gel bead as described below. The AGMs were washed with Tris–EDTA (TE) buffer (pH 7.4) to remove excess EDTA, suspended in a one-half volume of TE buffer, and stored at 4 °C. The entire process of the AGM preparation can be done in 7 h.

*Escherichia coli* DH5α was used as a model microorganism. *E. coli* cells were added at a concentration of 3 × 10^6^ cells mL^−1^ to alginate-CaCO_3_ solution and were encapsulated in 5.64 ± 1.72 × 10^5^ AGMs (mean ± SD) in three independent experiments, which corresponded to 12.5% ± 5.4% of the total AGMs. Of the AGMs containing *E. coli* cells, approximately 94% harboured a single cell in the core (Supplementary Table S1). In the three independent experiments, the diameters of the prepared AGMs were 39.6 ± 22.2 µm, 51.4 ± 13.3 µm and 49.3 ± 19.6 µm (Supplementary Fig. S3a). AGM diameters and core volumes showed normal distributions and log-normal distributions, respectively (Supplementary Figs. S3b, S4). The average median AGM core volume in the three experiments was 15.1 ± 6.3 pL (n = 3).

### MDA of single-cell genomes within AGMs

AGMs (in 50 µL suspension) were washed with sterile water and collected by centrifugation. The lysis of bacterial cells and denaturation of double-stranded DNA within the AGMs were carried out at room temperature for 30 min with 50 µL of an alkaline solution, which was Buffer D2 of the REPLI-g UltraFast Mini Kit (Qiagen). Denaturation was stopped by neutralisation with the addition of an equal volume of Stop solution from the kit, and the supernatant was then removed by centrifugation. Reaction buffer (93.5 µL) containing 11 µL of REPLI-g UltraFast DNA Polymerase from the kit was added, and the resulting mixture was incubated at 30 °C for 3 h. The reaction was stopped with one-tenth volume of 0.5 M EDTA, and the AGMs were washed three times with 200 µL of TE buffer. All the reagents used in MDA and the AGM preparation were irradiated with ultraviolet light to degrade any contaminating DNA prior to use.

**Figure 2:**
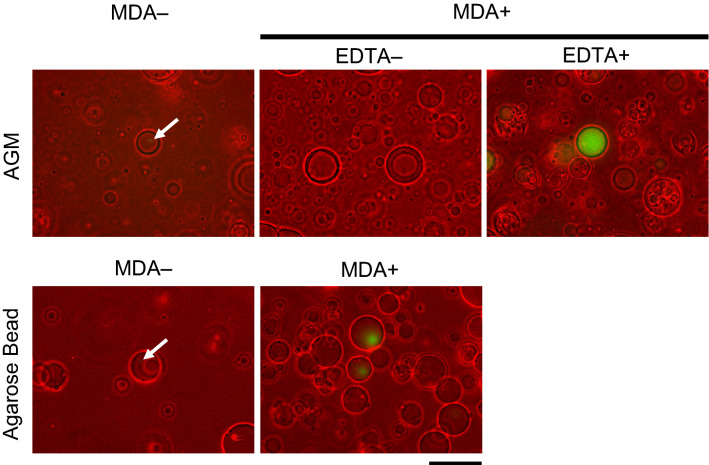
Yield enhancement of multiple displacement amplification (MDA) by alginate core solation. The effect of alginate core solation on the productivity of MDA within agarose gel microcapsules (AGMs) was evaluated. The genomic DNA of *Escherichia coli* encapsulated within AGMs was amplified by MDA with or without alginate solation using EDTA. The amplified DNA was detected with SYBR Green I. A phase contrast image (red) and epifluorescent image (green, SYBR Green I) are overlaid. MDA in agarose gel beads (without an alginate core), which was prepared using gelation of agarose droplets containing *E. coli* in oil emulsion, was also conducted as a control experiment. Arrows indicate *E. coli* cells stained with SYBR Green I. Bar = 100 µm.

Among the 4.6 ± 2.9 × 10^5^ AGMs prepared with *E. coli* cells, 8.9% ± 0.8% exhibited DNA amplification, which corresponded to 77.0% ± 27.2% of the AGMs containing *E. coli* cells (n = 3) (Supplementary Table S1). As shown in Fig. 2, amplified DNA filled the alginate sol core, whereas AGMs with alginate gel cores that were prepared without the solation step with EDTA or agarose gel beads containing no alginate core exhibited only limited or no DNA amplification by MDA.

### Quality of single-cell genome sequences compared between MDA-in-AGM and a conventional method using FACS-MDA

The quality of single-cell amplified genomes (SAGs) using MDA-in-AGM was evaluated and compared with that obtained by FACS-MDA. AGMs containing amplified DNA that was stained with SYBR Green I were individually transferred to 0.2 mL PCR tubes with 29 µL of sterile water under an epifluorescence microscope equipped with a TransferMan NK2 micromanipulator (Eppendorf), which required approximately 5 min per AGM. The micromanipulator was used in this study due to its low initial cost. Several tens of the picked AGMs were sequenced as follows. Amplified DNA in the AGM was released into water by heating the tubes at 60 °C for 5 min and directly used for the preparation of sequencing libraries using the QIAseq FX DNA Library Kit (Qiagen). No second round of MDA was necessary to obtain libraries suitable for genome sequencing. Paired-end sequence reads were generated on an Illumina MiSeq platform with the Reagent Kit V3 (600 cycles), and a fixed number of read pairs were randomly chosen and assembled de novo into contigs using SPAdes 3.13.0^28^ after standard quality filtering. Contigs > 1 kb were used for the subsequent analyses. Several factors describing the genome sequence quality, including completeness and contamination rate, were evaluated using CheckM^29^ and QUAST^30^ and compared between the SAGs obtained using MDA-in-AGM and FACS-MDA.

**Figure 3:**
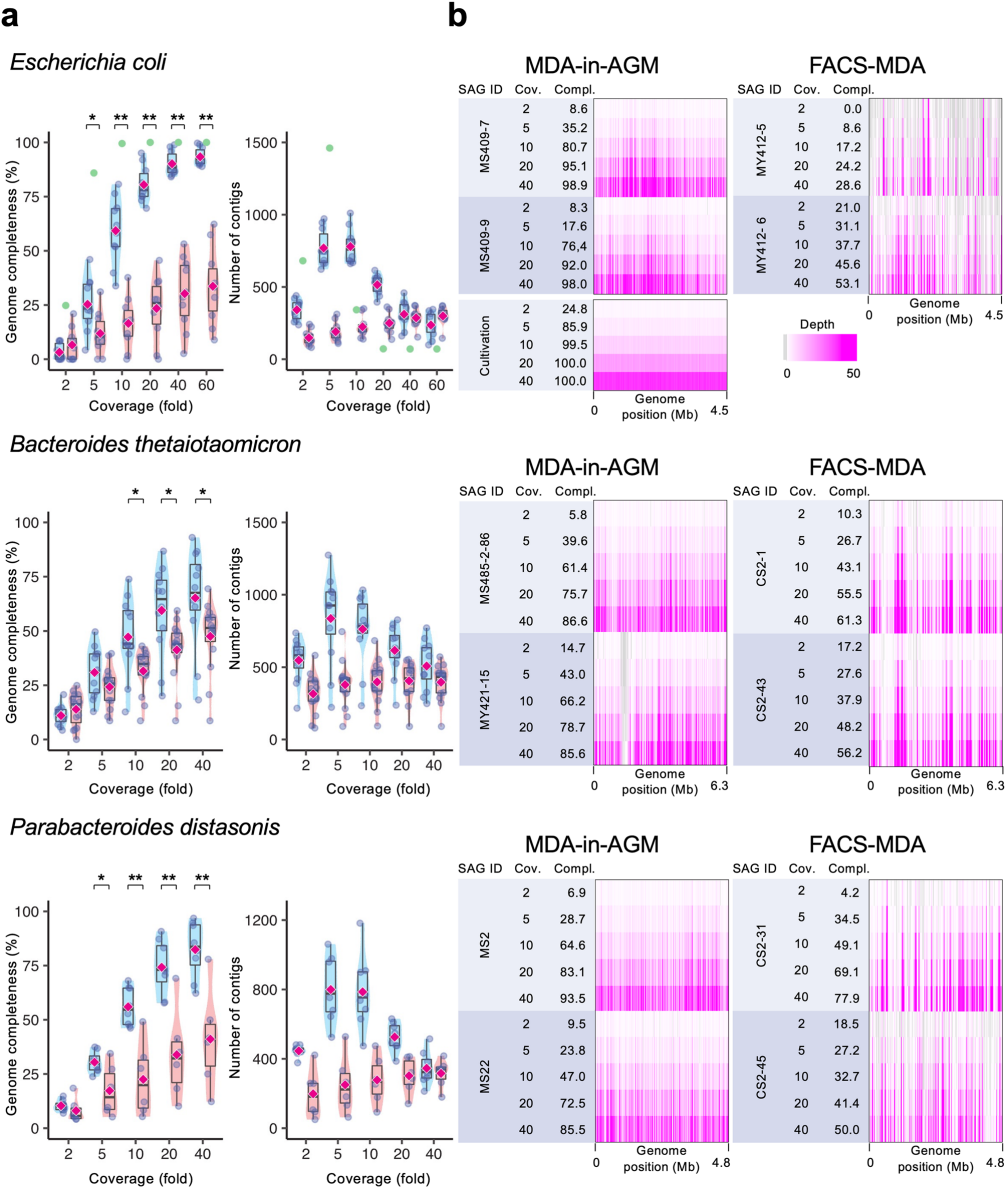
Comparison of genome completeness and amplification bias of single-cell genomes obtained by MDA-in-AGM and FACS-MDA. (**a**) Completeness and number of contigs of SAGs during the sequencing process. Box-and-violin plots (blue: MDA-in-AGM; red: FACS-MDA) are shown for *Escherichia coli* and two bacterial species as examples from the mock community of human gut bacteria (Supplementary Tables S2, S5 and S6). Datapoints (purple circles) and their arithmetic means (rhombuses) are shown on the plots. In the *E. coli* graphs, the results for DNA prepared from cultured *E. coli* cells without MDA are also shown (green circles). In the left panels, significant differences are indicated with asterisks (*P* < 0.05) or double asterisks (*P* < 0.01). (**b**) The number of sequence reads mapped against genome regions is shown as an indicator of amplification bias caused during MDA. The numbers of reads corresponding to different coverages used for de novo assembly (shown as ‘Cov.’) and genome completeness (%) (‘Compl.’) estimated using CheckM are shown.

The genome completeness and the total sequence length of *E*. *coli* SAGs obtained by MDA-in-AGM rapidly increased during the sequencing effort, and the average genome completeness reached 93.3% ± 4.1% (n = 10) for assemblies using 60-fold coverage, while it was 33.7% ± 17.3% (n = 10) in FACS-MDA (Student’s *t* test, *P* < 0.01) (Fig. 3a, Supplementary Table S2). The covered genome calculated by mapping reads to the reference *E*. *coli* DH5α genome sequence (BOCF01000000) was 97.4 ± 2.1% in MDA-in-AGM (n = 10), which was much higher than 47.8% ± 13.5% in FACS-MDA (n = 10) for 60-fold coverage reads (*P* < 0.01) (Supplementary Table S2). The steep decrease in the number of contigs, i.e. smooth assembly of contigs, in MDA-in-AGM along the sequencing effort’s depth was probably due to the lower amplification bias (Fig. 3a). In addition, the amplification bias among genome regions, which is inherent to MDA, was much improved in MDA-in-AGM compared to FACS-MDA (Fig. 3b). The amplification bias of SAGs was further evaluated using Gini coefficients and Lorenz curves, which indicate the population disparity as indices and plots. The Gini coefficients of the SAGs obtained by MDA-in-AGM were much lower than those of FACS-MDA (Welch’s *t* test, *P* < 0.01), indicating higher uniformity of the MDA-in-AGMs than the FACS-MDAs (Supplementary Fig. S5a). In the Lorenz curve, the SAGs obtained by MDA-in-AGM showed higher amplification uniformity than those by FACS-MDA (Supplementary Fig. S5b). Other quality metrics of SAGs using all sequence reads are shown in Supplementary Table S3.

### Single-cell genome analyses of a mock community and a natural sample

To evaluate the feasibility of MDA-in-AGM in a more realistic sample, we constructed a mock community comprising 15 cultured strains of human gut bacteria. The taxonomy and compositions of each bacterial strain estimated using 16S rRNA amplicon analysis or metagenome analysis are shown in Supplementary Table S4. SAGs from the mock community were obtained using either MDA-in-AGM or FACS-MDA in the same way as above. Taxonomic compositions of SAGs were similar between MDA-in-AGM and FACS-MDA, both of which successfully recovered SAGs of most strains accounting for > 5% of the mock community with the exception of *Faecalibacterium prausnitzii* (Supplementary Table S4). For example, SAGs of *Bacteroides thetaiotaomicron* and *Parabacteroides distasonis* exhibited clear differences between MDA-in-AGM and FACS-MDA in genome completeness, number of contigs, and amplification bias, as seen in the *E. coli* SAGs (Fig. 3, Supplementary Fig. S5). Since many SAGs obtained from environmental samples were difficult to compare at the same coverage due to lack of their reference genomes, the mock SAGs were assembled using 0.3 M read pairs in the following experiments. The overall genome completeness of SAGs with < 5% contamination was significantly higher in MDA-in-AGM (68.0% ± 23.3%, n = 39) than in FACS-MDA (42.4% ± 20.5%, n = 36) (*P* < 0.01) (Supplementary Tables S5, S6). The SAGs of most other strains also showed similar results to the *E. coli* SAGs (Supplementary Fig. S6).

**Figure 4:**
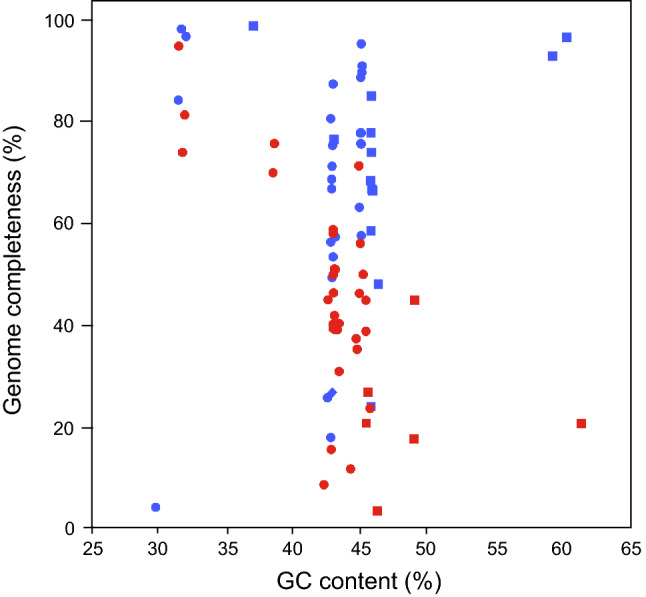
Relationships between genome completeness and GC contents of single-cell genomes in the mock community of human gut bacteria. Single-cell amplified genomes (SAGs) from the mock community comprising 15 strains of human gut bacteria are plotted with their GC contents against their genome completeness. The SAGs were assembled from 0.3 M randomly chosen read pairs, while MS485-1-51 of MDA-in-AGM (0.26 M read pairs) and CS1–94 of FACS-MDA (0.29 M read pairs) used all read pairs since they were fewer than 0.3 M. The bacterial species comprising the mock community and detailed results are shown in Supplementary Tables S4–S6. Blue and red indicate MDA-in-AGM and FACS-MDA, respectively. Squares, circles and rhombuses indicate gram-positive, gram-negative and gram-variable bacteria, respectively.

Most sequence reads were mapped to their respective reference genomes: the median mapping rates were 95.1% and 97.6% for SAGs from MDA-in-AGM and FACS-MDA, respectively (Supplementary Table S7). Although the rates of cross-contamination were slightly higher in MDA-in-AGM than in FACS-MDA, both were lower than 5% (Supplementary Table S7). In *Ba. thetaiotaomicron* SAGs, sequence reads from a *Ba. thetaiotaomicron* plasmid were also obtained (Supplementary Table S7). Our results indicated that MDA-in-AGM is applicable to various bacterial species and improves genome completeness in both gram-positive and gram-negative bacteria, regardless of their GC content (Fig. 4). For *Fa. prausnitzii*, it is possible that the cell lysis process using KOH was not effective, and hence no SAGs were obtained in either MDA-in-AGM or FACS-MDA.

SAGs from MDA-in-AGM with > 5% contamination, which would be caused either by contaminating extracellular DNA or by multiple cells being encapsulated in a single AGM, were binned into each bacterial species using metaWRAP^31^, although discrimination between contaminating DNA and DNA from captured multiple cells was difficult. As a result, 30 additional SAGs with < 5% contamination were obtained, and their genome completeness and contamination rates were 73.5% (median) and 0.9% (median), respectively (Supplementary Table S8).

DNA rearrangement during MDA, i.e. the formation of chimeras, can complicate the de novo assembly of single-cell genomes^32^. In *E. coli* SAGs, the rates of chimeric reads were slightly higher in MDA-in-AGMs (6.28% ± 1.09%) than in FACS-MDAs (4.49% ± 1.32%) (Welch’s *t* test, *P* < 0.01; Supplementary Table S3). In SAGs from the mock community, the rates of chimeric reads were higher in MDA-in-AGM (8.22% ± 2.28%) than in FACS-MDA (3.55% ± 1.07%) (Welch’s *t* test, *P* < 0.01; Supplementary Tables S5, S6). These rates of chimeric reads in MDA-in-AGM were similar to or slightly higher than the rate of 6.19% in a previous study using a conventional MDA method for sequencing human haploid genomes^33^.

Finally, we applied MDA-in-AGM to a natural environmental sample. We used the microbiota in the gut of the termite *Reticulitermes speratus*, which comprises several hundred bacterial species from diverse phyla^34^. The termite gut microbiota has attracted researchers especially for the complex symbiotic system enabling the highly efficient plant biomass degradation^35–37^. The termite gut microbiota is also superior for a benchmarking study using a natural environmental sample because its bacterial community structure is highly consistent within a termite species and has been well characterised^34,36,38,39^ and, in addition, the complete genome sequences of dominant bacterial species in *R. speratus* guts have been reported^40–42^.

The entire guts of five worker termites were removed, and the gut contents were suspended. Then, bacterial cells were collected at 18,000×*g* for 5 min by centrifugation after digesting the extracellular DNA with DNase I, and the cells were washed with sterile water as described previously^43^. The subsequent procedures were the same as those described above. To evaluate MDA-in-AGM using environmental specimens from the termite guts, we analysed 48 SAGs obtained by MDA-in-AGM. The SAGs showed a high genome completeness (78.6% ± 18.2%) and a low contamination rate (1.0% ± 1.0%) (Table 1). The SAGs were taxonomically classified using the Genome Taxonomy Database Tool Kit (GTDB-Tk)^44^ and were affiliated with 13 bacterial classes, including known dominant groups in the termite gut, Spirochaetia, Bacteroidia, Alphaproteobacteria, Betaproteobacteria, Deltaproteobacteria, Epsilonproteobacteria and Clostridia^34,38^. In contrast, the genome completeness of SAGs obtained by FACS-MDA was only 37.7% ± 19.0% (n = 161). Although a different sequencing method, i.e. a combination of the Nextera XT DNA Library Prep Kit (Illumina) and the Illumina HiSeq 2500 platform (500 cycles), was used for the latter case, the number of reads (bases) used for assembly was much larger on average (551 Mb ± 64 Mb, n = 161) than in the analysis of SAGs obtained by MDA-in-AGM (256 Mb ± 49 Mb, n = 48) (Supplementary Table S9).

**Table 1:** Summary of single-cell amplified genomes (SAGs) of symbiotic bacteria in termite gut bacteria.

Taxon of bacteria	MDA-in-AGM			FACS-MDA		
	Number of SAGs	Genome completeness (%)	Contamination (%)	Number of SAGs	Genome completeness (%)	Contamination (%)
Spirochaetia	15	73.5 ± 20.6	0.9 ± 1.1	35	32.5 ± 19.0	0.3 ± 0.6
Bacteroidia	8	88.2 ± 8.7	1.3 ± 0.7	51	36.1 ± 17.7	0.6 ± 1.2
Alphaproteobacteria	2	87.0 ± 1.3	0.0 ± 0.0	8	52.9 ± 19.5	0.9 ± 1.7
Betaproteobacteria	7	87.5 ± 5.9	1.2 ± 0.3	9	35.3 ± 12.6	0.9 ± 0.8
Deltaproteobacteria	3	86.4 ± 6.7	0.5 ± 0.3	3	22.8 ± 16.7	1.1 ± 0.6
Epsilonproteobacteria	2	55.0 ± 48.5	2.2 ± 3.1	2	38.8 ± 20.7	1.2 ± 0.6
Clostridia	4	70.2 ± 23.6	1.3 ± 0.4	27	36.7 ± 16.2	0.7 ± 1.1
Actinobacteria	1	51.9	0.87	3	33.5 ± 15.9	1.9 ± 1.7
Planctomycetales	1	87.1	0.1	11	42.6 ± 21.0	0.6 ± 0.8
Synergistia	2	83.9 ± 22.7	1.2 ± 1.6	4	65.5 ± 21.9	0.2 ± 0.3
Fibrobacteria	1	72.8	0.0	n. d.	n. d.	n. d.
Deferribacteres	1	72.9	2.0	n. d.	n. d.	n. d.
Candidate division SR1	1	74.1	0.0	n. d.	n. d.	n. d.
Bacilli	n. d.	n. d.	n. d.	8	47.2 ± 23.2	1.4 ± 2.0
Endomicrobia^†^	18	91.2 ± 9.8	1.5 ± 1.0	n. t.	n. t.	n. t.
Total sequenced samples	48 (66)	78.6 ± 18.2 (82.0 ± 17.2)	1.0 ± 1.0 (1.1 ± 1.0)	161	37.7 ± 19.0	0.7 ± 1.1

The numbers of SAGs, genome completeness and contaminants including Endomicrobia are shown in parentheses.

*n. d.* not detected, *n. t. *not tested.

^†^NGS libraries were made by Nextera XT and read by HiSeq.

In addition to the 48 SAGs, we analysed 18 SAGs of the class Endomicrobia with small genome sizes (~ 1 Mb)^40^ obtained by MDA-in-AGM, and 91.2% ± 9.8% genome completeness was obtained. Furthermore, among them, eight SAGs of phylotype Rs-D17 were compared to the complete genome sequences obtained previously^40,41^. The genome coverages calculated by mapping reads onto the two genomovars of Rs-D17 (Ri2008^40^ and Ti2005^41^) reached 95.7% ± 1.3% and 96.7% ± 0.9%, respectively (Supplementary Table S10). The composition of the termite gut community was estimated from SAGs obtained by MDA-in-AGM or 16S rRNA amplicon analysis (Supplementary Table S11). The taxonomic composition of the SAGs showed a high positive correlation (R = 0.834, *P* < 0.01; Pearson’s correlation coefficient) with that shown by the 16S rRNA amplicon analysis, whereas the frequencies of gram-positive bacteria were lower in SAGs.

## Discussion

MDA-in-AGM, which consisted of massively parallel MDAs in the picolitre-scale AGM cores, drastically improved the genome completeness of SAGs of *E. coli*, a mock community of human gut bacteria, and bacteria in the termite gut, compared to conventional microlitre scale FACS-MDA. Among them, the high-coverage SAGs obtained from the termite gut bacteria using MDA-in-AGM (Table 1, Supplementary Table S9) will prompt the understanding of the complex gut symbiotic system and the mechanisms of the highly efficient lignocellulose-decomposing system^34,36,40,41,43^. Thus, this method can reveal the metabolic capacity of yet-uncultured prokaryotes in environments such as animal intestinal tracts, soil and the hydrosphere.

The improvement of genome completeness in single-cell genomics by reducing the reaction volume of MDA using microfabrication techniques has previously been reported^10,15,17,20,45^, and the comparisons of MDA-in-AGM with those other MDA methods are shown in Supplementary Table S12. Although high genome completeness is also achieved using other miniaturized MDA methods, MDA-in-AGM does not require specialised equipment and provides scalable sample preparation. In addition, the use of alginate sol core successfully enhanced the yield of MDA, leading to the elimination of a second-round MDA step^20,45^ and hence leading to less amplification bias and simpler workflow. This high yield is probably attributable to the enhanced diffusivity of amplified DNA in the sol core^46^. Our MDA-in-AGM is applicable to both gram-positive and gram-negative bacteria and also to genomes with high GC content (Fig. 4). Additional advantages of the AGM are its physical stability and permeability to small molecules, which make buffer exchange and physical handling mush easier.

The formation of chimeric sequences during MDA can affect the efficiency and preciseness of the de novo assembly of SAGs^47^. In the mock community, the rates of chimeric reads were comparable with those in droplet MDA^19^ and slightly higher than in conventional MDA methods (Supplementary Table S12)^33^. Since the formation of chimeras is likely attributable to the branch migration of amplified DNA^32^, a higher MDA reaction temperature by using thermostable phi29 DNA polymerase^14^ may decrease erroneous hybridisation between replicons.

The uniformity of the alginate core sizes, which correspond to MDA reaction volumes (Supplementary Table S1), may have affected the yields of DNA-amplified AGM, the uniformity of the quantities of MDA products, and the quality of the AGM libraires. Although alginate cores with < 100-µm diameter were used for AGM preparations, a narrower size selection of the alginate cores, for example, 20–40 µm in diameter, would improve the uniformity of the AGM cores. Extrusion^48^ and microchannels^17–19^ would also improve the AGM core uniformity without size selection. However, introduction of these processes will complicate the workflow and increase the cost.

The manual picking procedure using a micromanipulator is the rate-limiting step in the single-cell genomics using MDA-in-AGM. To increase the throughput, AGM sorting using FACS or molecular barcoding^49^ of SAGs may be adapted, although these approaches also reduce the simplicity and inexpensiveness of the present protocol.

The taxonomic composition of SAGs obtained by MDA-in-AGM was largely consistent with those shown by 16S rRNA amplicon and metagenomic analyses; however, a SAG of the dominant *Fa. prausnitzii* was not recovered in either MDA-in-AGM or FACS-MDA (Supplementary Table S4). Thus, this failure was not specific to MDA-in-AGM but was attributable to the MDA procedure. Similar discrepancies in the taxonomic composition were found in gram-positive bacterial classes in experiments using termite gut microbiota (Supplementary Table S11), so modification of the cell lysis procedure, including the addition of lysozyme, may be required for certain gram-positive bacteria. Although we have not yet attempted, lysozyme with the size of 4 nm^50^ will diffuse through an agarose gel shell consisting of 2% low melting agarose (200-nm pore size)^51^ and lyse the bacterial cell wall in the core. To optimise such conditions, AGM is also superior because the exchange of the solution is easier compared to other methods.

The lower average genome completeness of the mock community (68%) and the termite gut microbiome (79%) compared to that of *E. coli* (93%) (Supplementary Tables S2, S5, Table 1) may be due to the differences in the ease of cell lysis. Low genome completeness of SAGs in certain bacterial lineages and environmental samples, compared to SAGs of cultured *E. coli*, has been reported in previous studies, irrespective of the MDA methods^1,52^. For example, in droplet single-cell MDA, the genome completeness of *E. coli* and soil microbiota has been found as 96.5% ± 2.2% (n = 16) and 52.8% ± 24.3% (n = 17), respectively^19^. Cell lysis optimisation for different prokaryotic strains and environmental samples is also important for increasing genome completeness.

Recently, as our experiments were on going, a report was published that shows MDA using a hollow-core hydrogel microcapsule consisting of a crosslinked PEG gel shell and a dextran sol core, a capsule structure similar to AGM^46^. Although amplification bias has not been evaluated, single-cell MDA in the core increases the amount of MDA product when compared with MDA in the gel bead. In addition, the shell of a hollow-core hydrogel microcapsule prevents genomic DNA from leaking from the core after alkaline denaturation prior to MDA. However, near-ultraviolet light (365 nm) used for photochemical crosslinking of the PEG shell damages cells and DNA due to photooxidation^53^. We selected agarose as the AGM shell because it allows thermally mild reversible gelation, is commercially available, and is stable in the presence of MDA reagents, especially alkaline solution, neutralisation buffer, and EDTA.

AGMs or other reaction chambers containing single bacterial cells inevitably produce a proportion of chambers containing multiple bacterial cells^21^, and it is difficult to completely eliminate contaminating DNA in our experimental procedures, as in other previously developed methods^1^. Nevertheless, the application of a computer program for binning metagenomic fragments, such as metaWRAP^31^, enabled us to recover a considerable number of at least median-quality SAGs from mini-metagenome bins that resulted from multiple cells by eliminating contaminating sequences (Supplementary Table S8).

In recent years, numerous studies analysing metagenome-assembled genomes from environmental samples have been reported^54^. Although single-cell genomics has different advantages and potentially increases the quality of research in combination with metagenomics, for example, by revealing strain-level heterogeneity^55^ and by providing information on horizontally transferred genetic components^45^, it is difficult for many microbiologists to perform. MDA-in-AGM is a much easier and less expensive method of single-cell genomics, and in addition, only a small amount of sample is needed. Thus, this method is also suitable for tiny specimens, such as the intestinal tracts of small insects and protist cells with endo- and ecto-symbiotic bacterial communities.

## Methods

### Reagents

Ultrapure DNase/RNase-Free Distilled Water (Thermo Fisher Scientific, Waltham, MA) was used for all solutions prepared in-house. The solutions were autoclaved at 121 °C for 15 min and further decontaminated by UV radiation at 7.2 mW/cm^2^ overnight (12 h) in a laminar flow cabinet.

A suspension of uniform CaCO_3_ nanoparticles (9.4% w/v, 100-nm diameter, pH 10) was provided by Shiraishi Central Laboratories (Hyogo, Japan). The conductivity and pH of the CaCO_3_ suspension were reduced by washing the particles using three volumes of water. In addition to Shiraishi's CaCO_3_, fine calcium carbonate such as precipitated calcium carbonate BioUltra (approximately 3-µm diameter; Sigma, St. Louis, MO) was also used for alginate core gelation. Alginate solution (5%, w/v) was prepared by dissolving sodium alginate (80–120 cP at 1%, w/v; Wako, Osaka, Japan) in water and stored at 4 °C. Agarose solution was prepared by dissolving SeaPlaque (final 2%, w/v; Lonza, Basel, Switzerland) in water and stored at 4 °C. Polyglyceryl-6 octacaprylate (PGO; Nisshin Oillio, Tokyo, Japan), Tris–EDTA buffer (TE; BioUltra for molecular biology, pH 7.4; Sigma), and REPLI-g UltraFast Mini Kit (Qiagen, Hilden, Germany) were used as described below.

### Evaluation of PGO for agarose microdroplet gelation

After an agarose solution (2 mL) was mixed with PGO or ISA, each containing 0.25% sorbitan monooleate (Span 85; Wako) (v/v) (20 mL, 35 °C), by vortexing and gelated on ice, agarose beads were recovered from PGO or ISA as described below. Large agarose aggregates were removed from the agarose beads using 300-µm cell strainers (pluriSelect, Leipzig, Germany). The agarose beads were observed under an inverted fluorescence microscope (IX71; Olympus, Tokyo, Japan) equipped with a CCD camera (BU-51LN; Bitran, Saitama, Japan). Diameters of the agarose beads over one pixel on the images (1.28 µm) were measured with ImageJ (https://imagej.nih.gov/ij/). The yields and diameters of the agarose beads were statistically analysed with R (https://www.r-project.org/).

### Preparation of Escherichia coli stock culture

*Escherichia coli* DH5α (TaKaRa Bio Inc., Shiga, Japan) from a single colony on an LB plate was cultured in two flasks with LB media (200 mL). *E. coli* cells were then harvested and washed twice in PBS (20 mL) by centrifugation. The cells were resuspended in PBS (10 mL), and their density was determined with a cell-counting chamber. The *E. coli* cells (3.05 × 10^10^ cell/mL)were divided into aliquots in microtubes (0.5 mL each) and stored at − 80 °C. As mentioned below, single cells of *E. coli*, the mock sample, and the termite gut microbiome were analysed using cells stored at − 80 °C, a mixture of glycerol stocks stored at − 80 °C, and live specimens from termite guts, respectively.

### Preparation of alginate cores containing *E. coli* cells

*Escherichia coli* cells were encapsulated in alginate cores by the emulsification/internal gelation method^27^ (Fig. 1b). *E. coli* cells from the stock culture (10 µL) were mixed with 990 µL of 2% sodium alginate solution containing 1% CaCO_3_ and 50 mM acetate buffer (pH 7.0, Sigma). The mixture was emulsified with 9 mL of isostearyl alcohol (ISA; Kokyu Alcohol Kogyo Co., Ltd., Chiba, Japan) containing 3% lecithin (Wako) in a 50-mL tube by vortexing for 1 min. The emulsion was mixed with 2% acetic acid in ISA (0.1 mL each) by vortexing ten times for 1 min each. During this procedure, the pH of the mixture gradually decreased to 4.0 and the CaCO_3_ nanoparticles were dissolved. The released calcium ions from CaCO_3_ gelated the alginate microdroplets in the emulsion. ISA was removed by mixing with 9 mL of diethyl ether and centrifuging at 4 °C for 3 min using a swing rotor (3000×*g*, 5702R, Eppendorf, Hamburg, Germany). The residual ISA was further removed from the alginate cores by mixing with 50 mM Tris–HCl buffer (pH 7.5) containing 0.2% (v/v) Tween 20 (Tris-Tween 20) (5 mL), followed by centrifugation, and then by repeating the procedure twice with 1-butanol (5 mL). The resulting alginate cores were suspended in one volume of 50 mM Tris–HCl (pH 7.5) and filtered through a 100-µm cell strainer (pluriSelect) to remove large alginate aggregates. The filtrated alginate cores were further washed with Tris–HCl (pH 7.5) and collected in 2-mL microtubes. The volume of the alginate cores was calculated from its weight and stored at 4 °C after mixing with a one-half volume of Tris–HCl (pH 7.5). To adjust the number of *E. coli* cells in a single alginate core to less than two, 10 µL of 3.05 × 10^6^–10^8^ *E. coli* cells from the *E. coli* stock were mixed with 990 µL of the alginate containing CaCO_3_ suspension. After alginate cores containing *E. coli* were prepared from the mixture, *E. coli* in the alginate cores were observed using SYBR Green I (TaKaRa Bio Inc.) (Supplementary Fig. S7). At 3.05 × 10^6^ cells/mL of the alginate mixture, 17.6% of the cores contained *E. coli*, and 78.8% of those carried single cells. Thus, 3.05 × 10^6^ cells/mL of *E. coli* was used for alginate core preparation in subsequent experiments.

### Preparation of AGMs containing *E. coli* cells

The supernatant of the alginate core suspension (600 µL) was removed after centrifugation in a 50-mL tube. The residual alginate cores (400 µL) were mixed with the agarose solution as prepared above (2 mL). The mixture was emulsified with 0.25% (v/v) Span 85 in PGO (20 mL) by vortexing for 1 min (Fig. 1b). The alginate cores, the agarose solution, and the PGO were preincubated at 35 °C for 10 min to prevent the formation of gelated large agarose aggregates. Then, the agarose was gelated by cooling on ice for 1 h. PGO was removed from the emulsion by washing as described above. The alginate gel core was solated by chelating calcium ions with the addition of a 1/10 volume of 0.5 M EDTA (Thermo Fisher Scientific). The AGMs were then suspended in one volume of TE buffer. Large debris in the suspension was removed through a 300-µm cell strainer, and the AGMs were further washed with TE. The AGMs were diluted in 50 mM Tris–HCl (pH 7.5) containing 0.5% SeaPlaque agarose, 0.1% *p*-phenylene diamine (Wako), and 10,000-fold diluted SYBR Green I (TaKaRa Bio Inc.), and the morphology, density, diameter, and number of encapsulated *E. coli* cells were observed as mentioned above. The AGMs were stored at 4 °C. For long-term storage (more than a week), the AGMs were stored in 40% ethanol at − 80 °C and washed three times in ten volumes of water before use. The quality of genomic DNA of AGMs stored in 40% EtOH was confirmed via their amplification using MDA.

### Evaluation of alginate core solation in AGM

Alginate gel cores without *E. coli* were labelled with rhodamine 123 (Wako) using 1-ethyl-3-(3-dimethylaminopropyl) carbodiimide hydrochloride (Tokyo Chemical Industry Co., Ltd., TCI, Tokyo, Japan) and *N*-hydroxysuccinimide (Wako)^56^. AGMs containing rhodamine 123-labelled cores were heated at 65 °C for 5 min in the presence of 50 mM EDTA or 50 mM CaCl_2_. Residual cores were observed as mentioned above.

### Effect of alginate core gelation and solation on MDA

Aliquots (50 µL) of the AGMs containing *E. coli* cells before solation of the alginate cores were mixed with one volume of cell lysis solution containing 400 mM KOH with or without 10 mM EDTA and they were neutralised with 100 µL of Stop buffer from the REPLI-g UltraFast Mini Kit. The AGMs containing *E. coli*, AGMs without *E. coli* (negative control), and agarose beads containing *E. coli* (control) were subjected to MDA as described below, and their amplified DNAs were observed.

### MDA-in-AGM

A 50-µL aliquot of AGMs was centrifuged, and the collected AGMs were washed with 200 µL water. Cell lysis and DNA denaturation were carried out with Buffer D2 (50 µL) of the REPLI-g UltraFast Mini Kit at room temperature for 30 min. After denaturation was stopped with the Stop buffer (50 µL), the supernatant was removed by centrifugation. The Reaction buffer (93.5 µL) containing 11 µL phi29 DNA polymerase from the kit was added and incubated at 30 °C for 3 h (MDA-in-AGM). The reaction was stopped with 1/10 volume of 0.5 M EDTA, and the AGMs were washed with 200 µL of TE buffer three times. The washed AGMs were stored at 4 °C.

### FACS-MDA

Single-cell MDA using a MoFlo XDP fluorescence-activated cell sorter (Beckman Coulter Inc., Brea, CA) was performed as a control^43^. After *E. coli*, the mock bacterial community and termite gut bacteria were stained with SYBR Green I or CellTracker Green (Thermo Fisher Scientific), and the stained bacteria were sorted into a single cell per well of 96-well PCR plates. Cell lysis was carried out with Buffer D2 (1.5 µL) of the REPLI-g UltraFast Mini Kit at room temperature for 30 min. After denaturation was stopped with the Stop buffer (1.5 µL), Reaction buffer (7.5 µL) containing 0.5 µL phi29 DNA polymerase from the kit was added and incubated at 30 °C for 3 h and 65 °C for 15 min. To check genome amplification and the degree of contamination, the MDA products were evaluated by direct Sanger sequencing of the 16S rRNA. PCR was performed with Bacteria-universal 16S rRNA gene primers: 27F (5′-AGAGTTTGATCMTGGCTCAG) and 1390R (5′-ACGGGCGGTGTGTACAA).

### Mock community of human gut bacteria

A mock community of human gut bacteria was prepared using 15 isolates (Supplementary Table S4) obtained from the Japan Collection of Microorganisms. A mixture of all glycerol stocks in equal amounts was used as the mock bacterial community. After the mock bacterial community was diluted 100-fold using water, the diluent (10 µL) was treated with DNase I (20 µL) at 30 °C for 30 min. The cells were washed three times by suspending in water (1 mL), collected by centrifuging at 18,000×*g* for 5 min, and suspended in water (10 µL). After the MDA-in-AGM as described above, the AGMs were observed with SYBR Green I using a microscope. Among the large numbers of AGMs in the microscopic field of view, only a few AGMs contained amplified DNAs, which were isolated using a micromanipulator as described below.

To determine the composition of the mock bacterial community, the total DNA was extracted using the DNeasy Ultra Clean Microbial Kit (Qiagen). Metagenome sequencing was performed by preparing a library with the QIAseq FX DNA Library Kit (Qiagen). Amplicon sequencing of 16S rRNA genes in the mock community was also performed by preparing a library with the Nextera XT Index Kit 96 indices (Illumina, Inc., San Diego, CA) and the MiSeq platform with Reagent Kit V3 (600 cycles). The bacterial composition of the mock community was estimated on the basis of the results analysed in QIIME2^57^.

### Termite gut bacteria

Specimens of the wood-feeding termite *Reticulitermes speratus* were collected in Tsukuba in Ibaraki Prefecture, Japan. The guts of five worker termites were removed, and the gut contents were suspended in solution U^58^, which consisted of 9.2 mM NaHCO_3_, 5.1 mM trisodium citrate dihydrate, 13 mM KH_2_PO_4_, 37 mM NaCl, 0.75 mM CaCl_2_ and 0.4 mM MgSO_4_. The extracellular DNA and protist DNA were digested using DNase I, and the bacterial cells were collected at 18,000×*g* for 5 min by centrifugation and washed with water as mentioned above^43^. The washed termite gut bacteria were suspended in water (10 µL). MDA-in-AGM for the termite gut bacteria was performed using the suspension as the mock community.

### Genome sequencing and bioinformatics

AGMs containing amplified DNA, detected with SYBR Green I, were transferred individually to PCR tubes under an inverted fluorescence microscope equipped with a micromanipulator (TransferMan NK2; Eppendorf, Hamburg, Germany). Water (29 µL) was added to each PCR tube with a single AGM and incubated at 60 °C for 5 min to release DNA by solating the agarose shell. To check the degree of contamination, we screened the MDA-in-AGM products by direct Sanger sequencing of 16S rRNA gene as mentioned above. After removing the contaminated samples, sequencing libraries were prepared using the QIAseq FX DNA Library Kit. Since the MDA products in AGM cores were too small to quantify, the fragmentation and ligation times in the library construction were performed using a condition of the < 10 ng input DNA in the manufacturer’s instructions. Genome sequencing was performed on the MiSeq platform with Reagent Kit V3 (600 cycles). Sequence libraries for single cells isolated from the termite gut microbiota using FACS were prepared using the Nextera XT DNA Library Prep Kit (Illumina) and analysed on the Illumina HiSeq 2500 platform (500 cycles).

The generated short reads were trimmed using Cutadapt (https://github.com/marcelm/cutadapt), PRINSEQ (http://prinseq.sourceforge.net/), FASTX Trimmer, FASTQ Quality Trimmer (http://hannonlab.cshl.edu/fastx_toolkit/download.html) and cmpfastq_pe (http://compbio.brc.iop.kcl.ac.uk/software/download/cmpfastq_pe). The trimmed reads were assembled using SPAdes ver. 3.13.0 (k-mer: 21, 33, 55, 77, 99 and 127, options: -sc, -careful)^28^ into contigs. Only > 1 kb contigs were selected using the SeqKit (https://bioinf.shenwei.me/seqkit/) for the subsequent analyses.

Taxonomic classification of single-cell genomes from termite gut samples was conducted using the Genome Taxonomy Database Tool Kit (GTDB-Tk)^44^. Genomic sequence data showing > 5% contamination, identified using CheckM^29^, were sequentially subjected to binning, refinement, and reassembly processes with the BINNING (including metaBAT2^59^, MaxBin2^60^ and CONCOCT^61^), BIN_REFINEMENT, and REASSEMBLE_BINS modules of metaWRAP^31^.

For single-cell genome analyses using *E. coli* DH5α and human gut bacteria with increasing coverage (Fig. 3), adapter-trimmed reads using Trimmomatic^62^ were randomly chosen using Rasusa^63^ and assembled de novo using SPAdes^28^. Genome completeness and numbers of contigs in the assemblies were evaluated using CheckM^29^ and plotted using R. The sequencing reads corresponding to different coverage were mapped onto known genome sequences using Bowtie2^64^, and the results were visualised as heatmaps using IGV ver. 2.3.26 (http://software.broadinstitute.org/software/igv/). Covered genome (%), which was the genome regions covered with at least one read per 1 kb-bin, was calculated using BBtools (http://jgi.doe.gov/data-and-tools/bbtools/).

The amplification biases of SAGs were evaluated using Gini coefficients and Lorenz curves. After sequencing reads of SAGs were mapped on reference genomes using Bowtie2^64^, the reads were randomly chosen to be 60-fold (*E. coli*) and 40-fold (human gut bacteria) coverage of their genomes, and sequencing depths per base were calculated from the reads using SAMtools (http://www.htslib.org). Gini coefficients of SAGs were calculated from mean sequencing depths per 50-kb bin using the R ineq package. SAGs, which had Gini coefficients equivalent to their medians, were further plotted on Lorenz curves using the R gglorenz package.

The chimeric read rate was calculated by mapping short reads to the reference genome with the Barrows-Wheeler Aligner (https://sourceforge.net/projects/biobwa/files/) and SAMtools. The reference genomes are listed in Supplementary Table S4. Cross-contaminations of genomic sequencing data with < 5% contamination using CheckM^29^ (Supplementary Table S7) were calculated by mapping sequencing reads to reference genomes in the mock community^16^.

## Supplementary Information

Supplementary Tables.

Supplementary Figures.

## Data Availability

The raw fastq files (DRR253532–DRR253885) and assemblies for SAGs of termite gut bacteria (BNTM01000000–BOCE01000000) were deposited into DDBJ under BioProject Accession No. PRJDB10679.
