# Supplementary material for: Agarose gel microcapsules enable easy-to-prepare, picolitre-scale, single-cell genomics, yielding high-coverage genome sequences
Source: Sci Rep. 2022 Oct 18;12:17014. doi: 10.1038/s41598-022-20923-z (PMC9579161; doi:10.1038/s41598-022-20923-z)
Supplement: Supplementary file 2 — Supplementary Figures. [file 41598_2022_20923_MOESM2_ESM.docx]

## Supplementary Information

### Figures

### Fig. S1 Polyglyceryl-6 octacaprylate (PGO), an oil with water-equivalent density, stably gelated agarose microdroplets in an emulsion.

PGO, an oil with water-equivalent density (0.997 g/mL), was tested as an emulsion oil for its capability to stably gelating agarose shells by preventing aggregation. By using agarose sol droplets instead of agarose sol shells in the agarose gel microcapsules (AGM), PGO was tested for (**a**) water-in-oil emulsion stability, (**b**) the degree of aggregation of agarose gel droplets, (**c**) diameter, (**d**) shapes, and (**e**) yield of agarose gel droplets. Isostearyl alcohol (ISA; density 0.836 g/mL) was used as a conventional emulsification oil for comparison.

**a.** Emulsion stabilities formed by water and PGO (right) or water and ISA (left). The emulsion was observed at 0, 30, and 60 min after 0.5 mL of Milli-Q water (Millipore) and 0.5 mL of 3% lecithin in ISA or PGO were mixed by vortexing for 1 min.

**b.** Aggregation of agarose gel droplets in the PGO (left) and ISA (right) emulsions.

**c.** Diameter of agarose gel droplets formed in the PGO (white) and the ISA (grey) emulsions. Values of three independent experiments are shown as box-and-violin plots using the R ggplot2 package (https://ggplot2.tidyverse.org/index.html). Rhombuses (red) and circles (black) denote arithmetic means and outliers, respectively. The numbers of agarose gel droplets used for the diameter measurement are shown in parentheses. Double asterisks indicate significant differences (Wilcoxon rank sum test, *P* < 0.01).

**d.** Shapes of agarose gel droplets formed in the PGO (left) and ISA (right) emulsions.

**e.** Yields of agarose gel droplets with <300 µm diameter from 2 mL of agarose sol solution gelated in the PGO or ISA emulsions. Values are shown as the mean ± SD. An asterisk indicates a significant difference (n = 3, Welch's *t*-test, *P* < 0.05).

### Fig. S2 Evaluation of the sol state of AGM alginate cores.

By dissolving agarose shells, alginate cores in AGMs were examined for whether they were sol or gel. AGMs containing rhodamine 123-labelled alginate cores were prepared, solated with EDTA, and observed under a microscope (before heating). The AGMs were then heated in the presence of EDTA or CaCl_2_, and their core diffusions were observed (after heating). A phase contrast image (red) and an epifluorescent rhodamine 123 image (green) are overlaid. The observation was done in different magnification (upper and bottom).

### Fig. S3 Diameter of AGMs.

**a.** Diameters of AGMs in three independent experiments are shown as box plots. The numbers of AGMs used for the measurement are shown in parentheses.

**b.** The AGM diameters in the three experiments are shown as histograms with estimated density functions of normal distributions.

### Fig. S4 Volume of AGM cores.

**a.** Volumes of AGM cores in three independent experiments are shown using box plots. The numbers of AGM cores used for the measurement are shown in parentheses.

**b.** Histograms of the AGM core volumes using linear scales with log-normal distribution curves (upper panels) and using logarithmic scales with normal distribution curves (lower panels).

### Fig. S5 Gini coefficients and Lorenz curves of single-cell amplified genomes obtained by MDA-in-AGM and FACS-MDA.

**a.** Gini coefficients of SAGs obtained by MDA-in-AGM (white) or FACS-MDA (grey) of three bacterial species in **Fig. S3a**. The Gini coefficients were calculated from mean sequencing depths per 50-kb bin through their genomes using R software. The numbers of SAGs used for the analysis are shown in parentheses. A lower Gini coefficient indicates a lower amplification bias. Double asterisks indicate significant differences (Welch's *t*-test, *P* < 0.01).

**b**. Lorenz curves of SAGs obtained by MDA-in-AGM (blue) or FACS-MDA (red). Sequencing depths per bin of representative SAGs in **Panel a**, which correspond to the median values in their Gini coefficients, are plotted into the Lorenz curves using R. Equally distributed lines (dashed) and the result from *E. coli* culture without MDA (green) are also plotted. In a Lorenz curve, the data of SAGs with a lower amplification bias approaches the equally distributed line.

### Fig. S6 Number of sequence reads mapped against genome regions.

The read coverage pattern is shown as an indicator of amplification bias caused during MDA. Randomly chosen reads (0.3 M read pairs) from SAGs were mapped to the corresponding reference genome sequences. 'Comp.' indicates the genome completeness (%) estimated using CheckM.

### Fig. S7 Optimisation of *E. coli* density

A series of diluted *E. coli* cells (0 to 3.05 × 10^8^ cell/mL alginate mixture) was used for the preparation of alginate cores. A phase contrast image (red) and an epifluorescence SYBR Green I image (green) are overlaid. In the panel of 3.05 × 10^6^ cells/mL, arrows indicate *E. coli* cells.


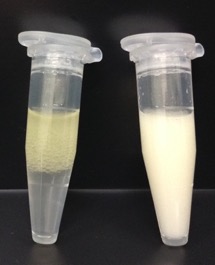

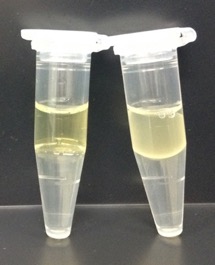

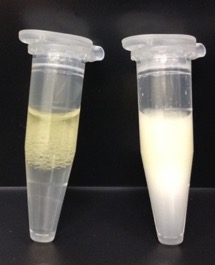

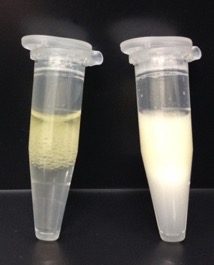


Before

After

0 min

30 min

60 min


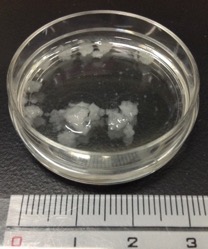

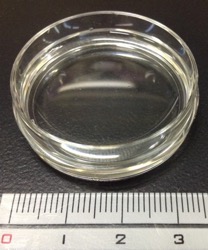


**b**

**c**


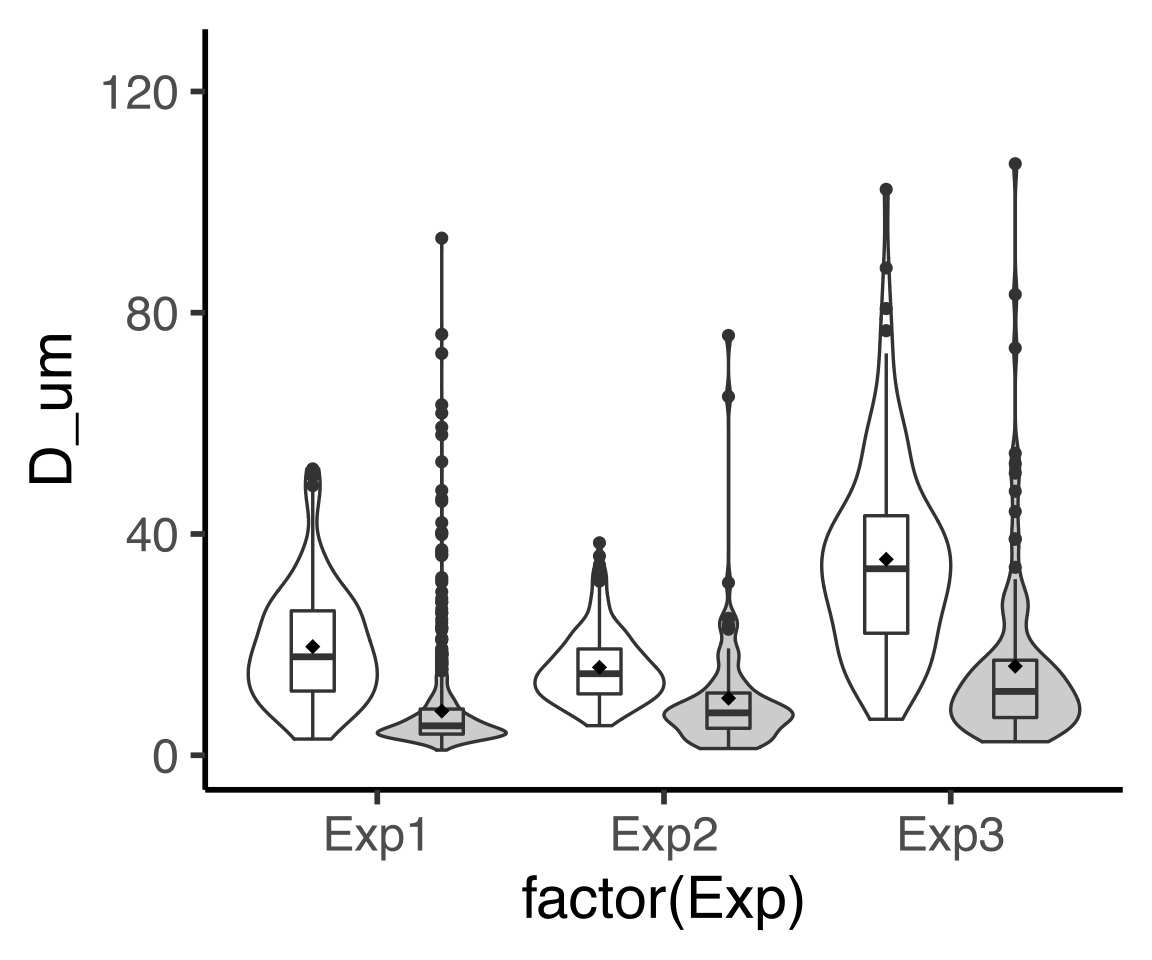


0

40

80

Diameter (µm)

120

(103)

(696)

(306)

(87)

(111)

(116)

No. 1

No. 2

No. 3

Fig. S1


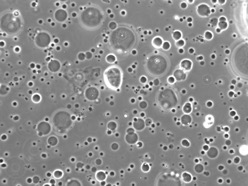

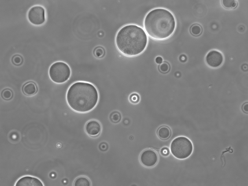


**d**


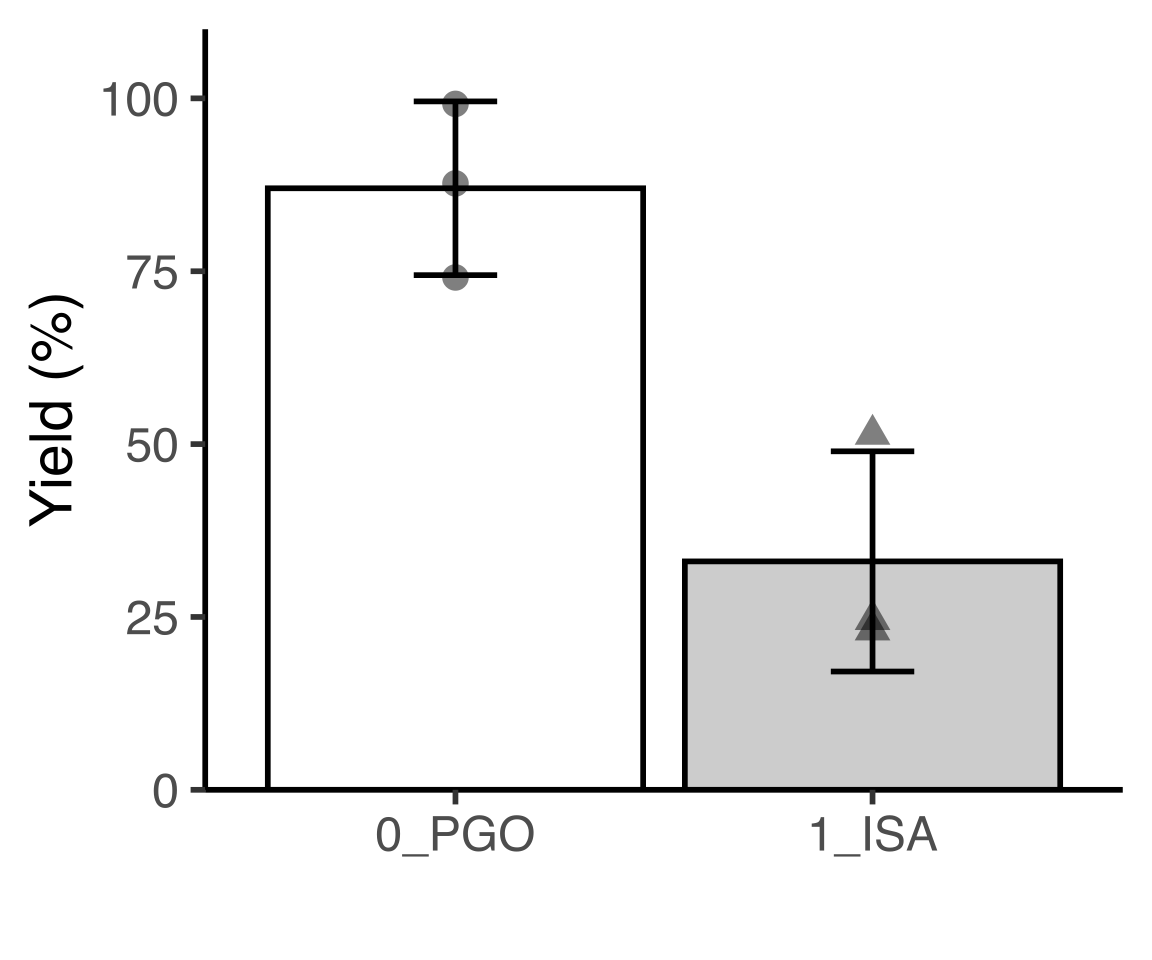


0

100

25

50

75

PGO

ISA

Yield (%) (%)

**e**

*

**aaaaaaaaaaaaaaaaaaaaaaaaaaaaaaaaaaaaaaaaaaaaaaaaaaaaaaaaaaaaaaaaaaaaaa

**aaaaaaaaaaaaaaaaaaaaaaaaaaaaaaaaaaaaaaaaaaaaaaaaaaaaaaaaaaaaaaaaaaaaaa

**aaaaaaaaaaaaaaaaaaaaaaaaaaaaaaaaaaaaaaaaaaaaaaaaaaaaaaaaaaaaaaaaaaaaaa

100 µm

**a**


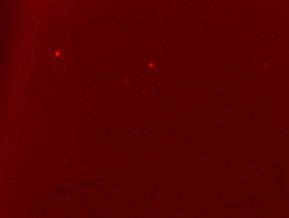

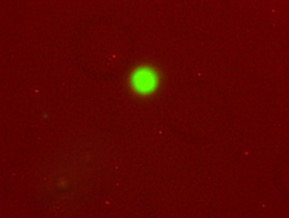

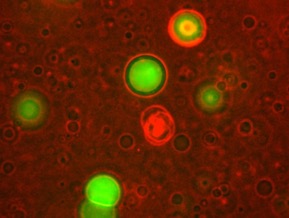

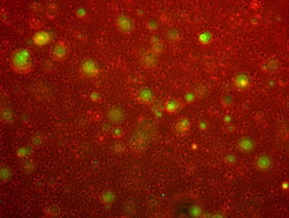

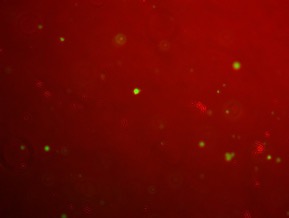

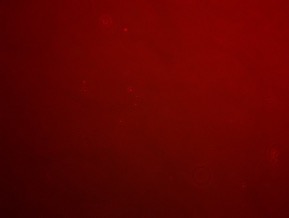


Fig. S2

100 µm

500 µm

Before Heating

After Heating

50 mM EDTA

50 mM CaCl_2_

**a**

Diameter (µm)

150

100

50

0

No. 1

No. 2

No. 3


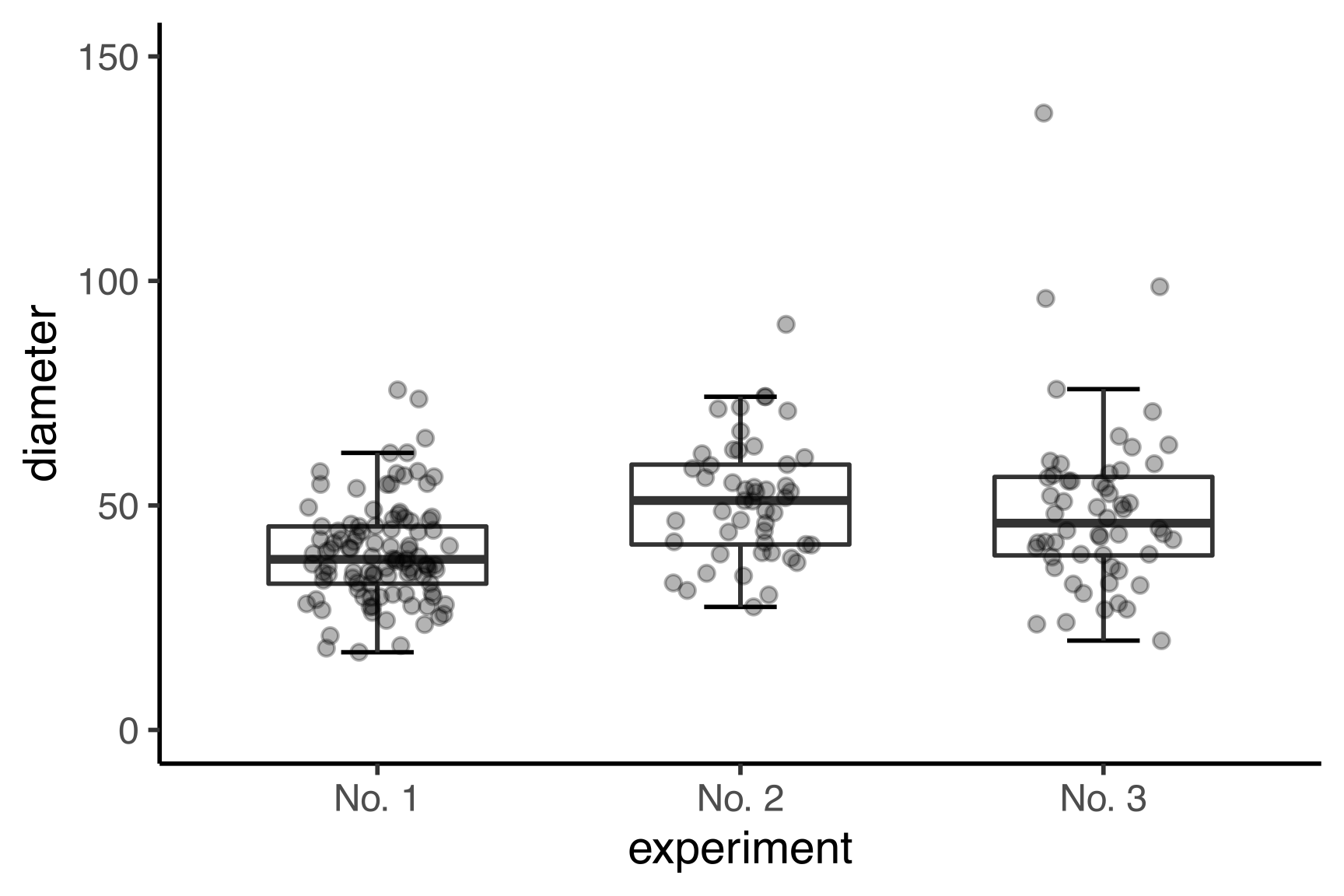


(104)

(49)

(56)

Experiment

**b**

Experiment

Diameter (µm)


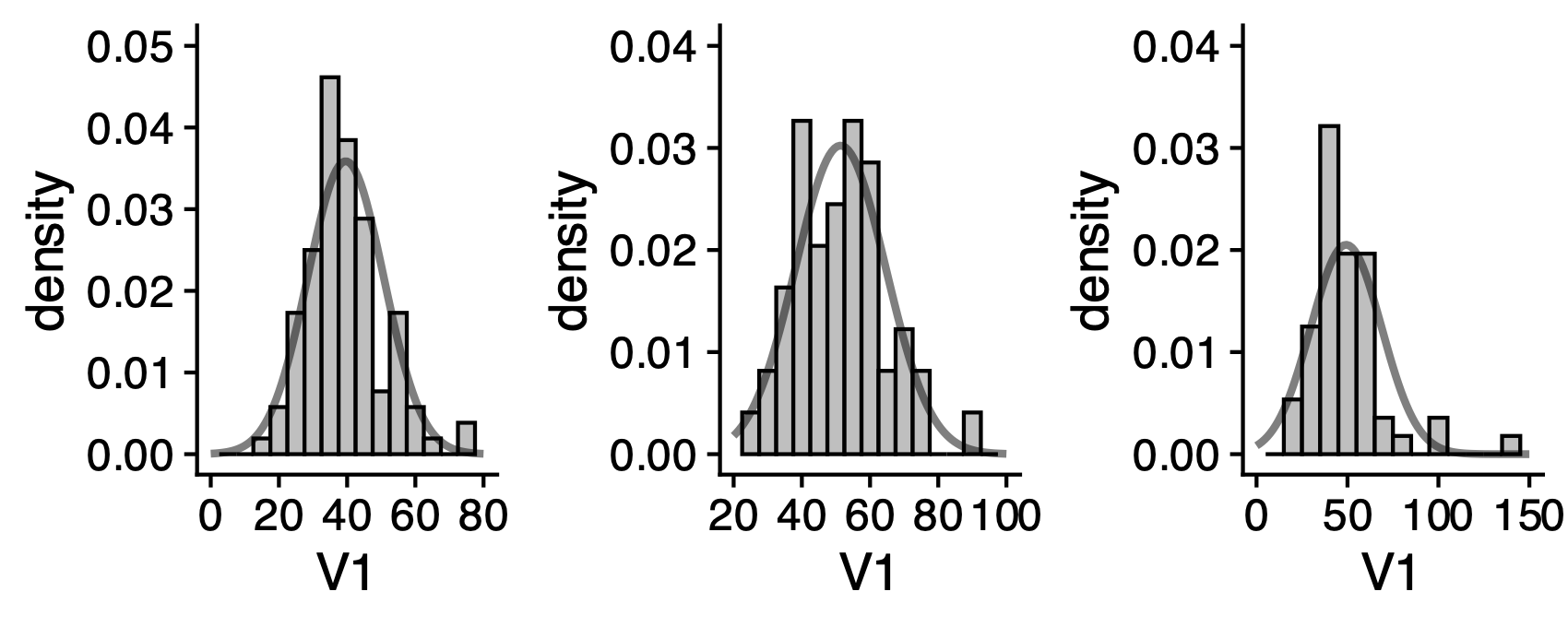


0.01

0.00

0.02

0.03

0.04

0.05

0.01

0.00

0.02

0.03

0.04

0.01

0.00

0.02

0.03

0.04

Density

No. 1

No. 2

No. 3

Fig. S3

0

20

40

60

80

0

20

40

60

80

100

50

100

150

**b**


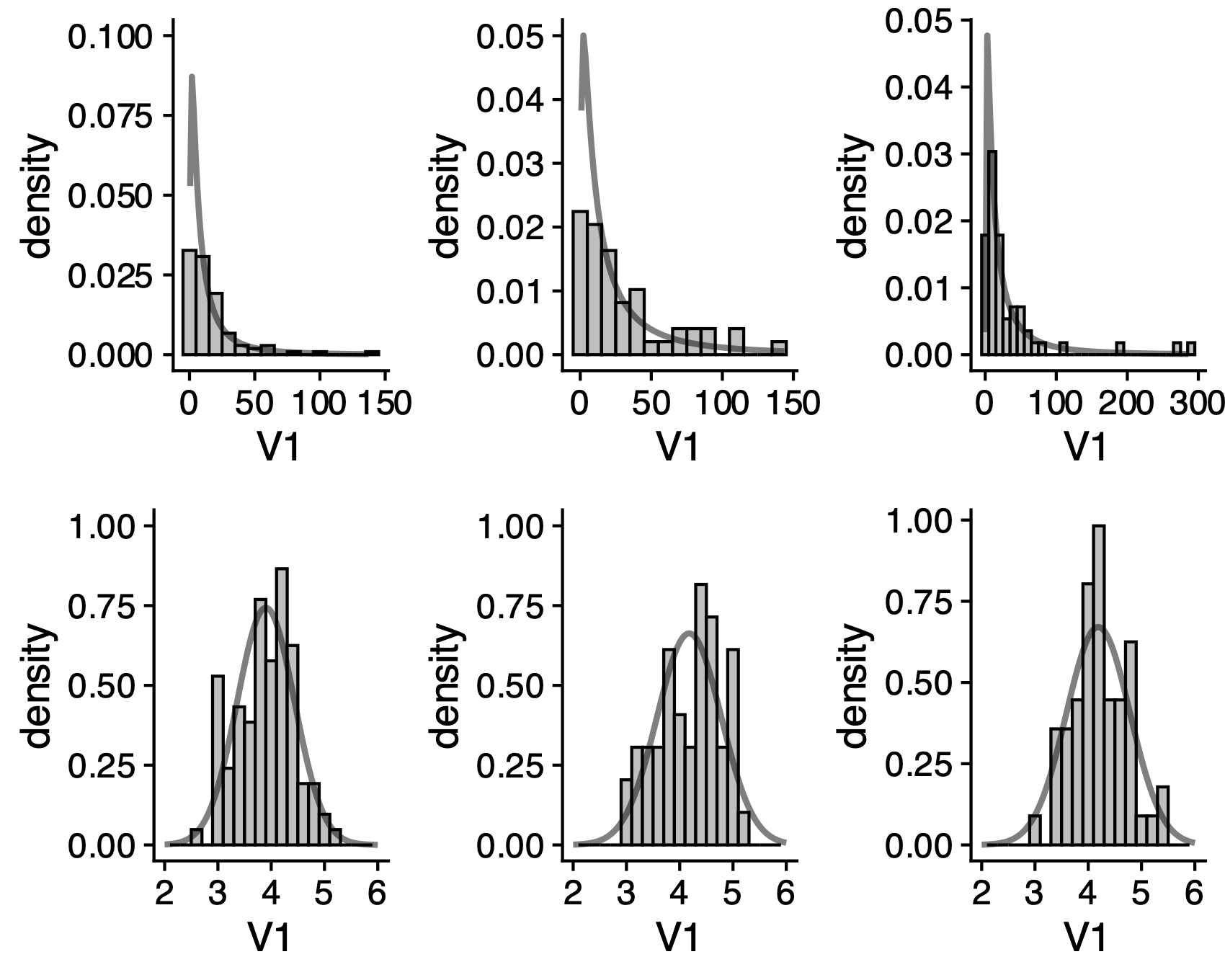


0.00

0.01

0.02

0.03

0.05

0.04

0.00

0.01

0.02

0.03

0.05

0.04

0.000

0.025

0.050

0.075

0.100

0

100

200

300

0

50

100

150

0

50

100

150

Core (pL)

Density

No. 1

No. 2

No. 3

0.00

0.25

0.50

0.75

1.00

0.00

0.25

0.50

0.75

1.00

0.00

0.25

0.50

0.75

1.00

Density

log_10_ [Core (fL)]

2

3

4

5

6

2

3

4

5

6

2

3

4

5

6

**a**


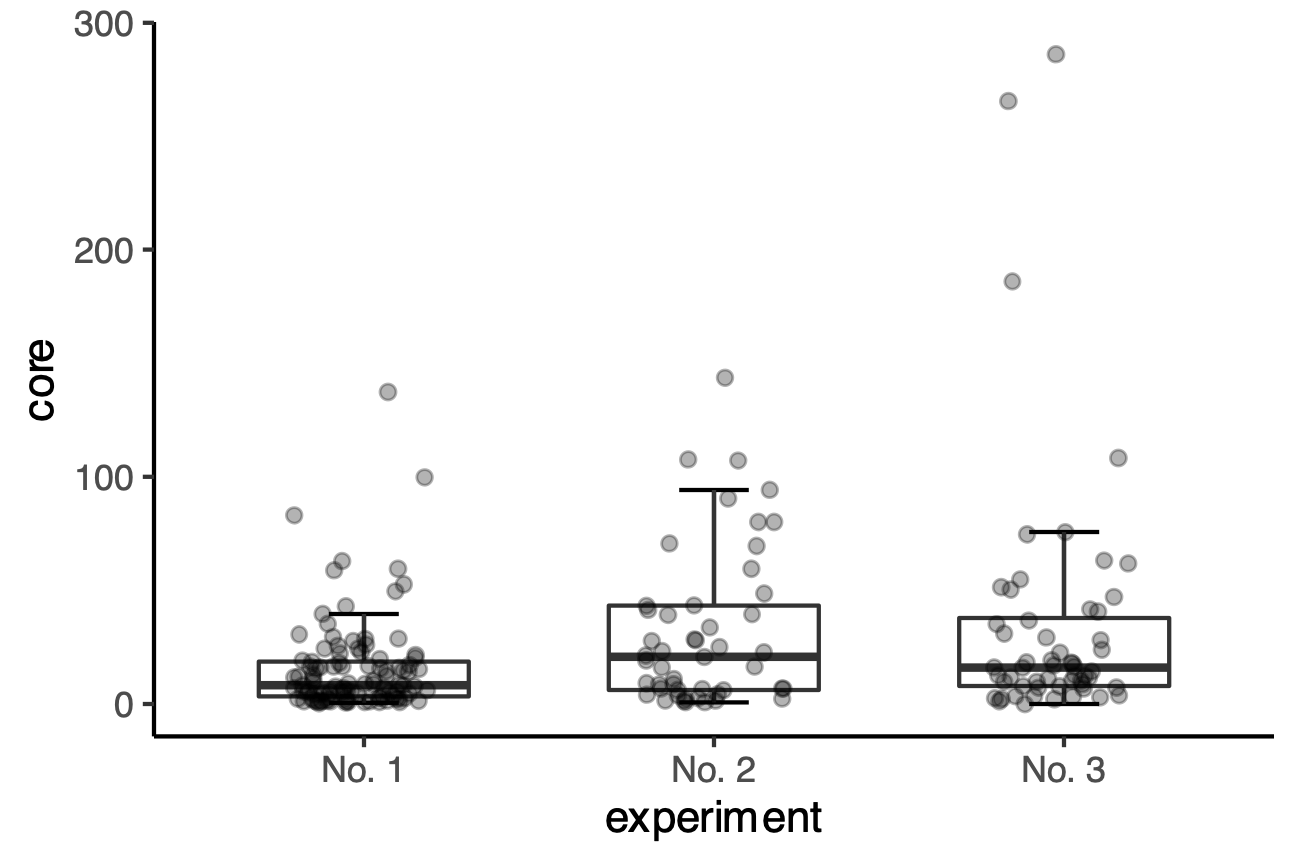


Core (pL)

No. 1

200

300

0

No. 2

No. 3

100

Fig. S4

(104)

(49)

(56)

Fig. S5


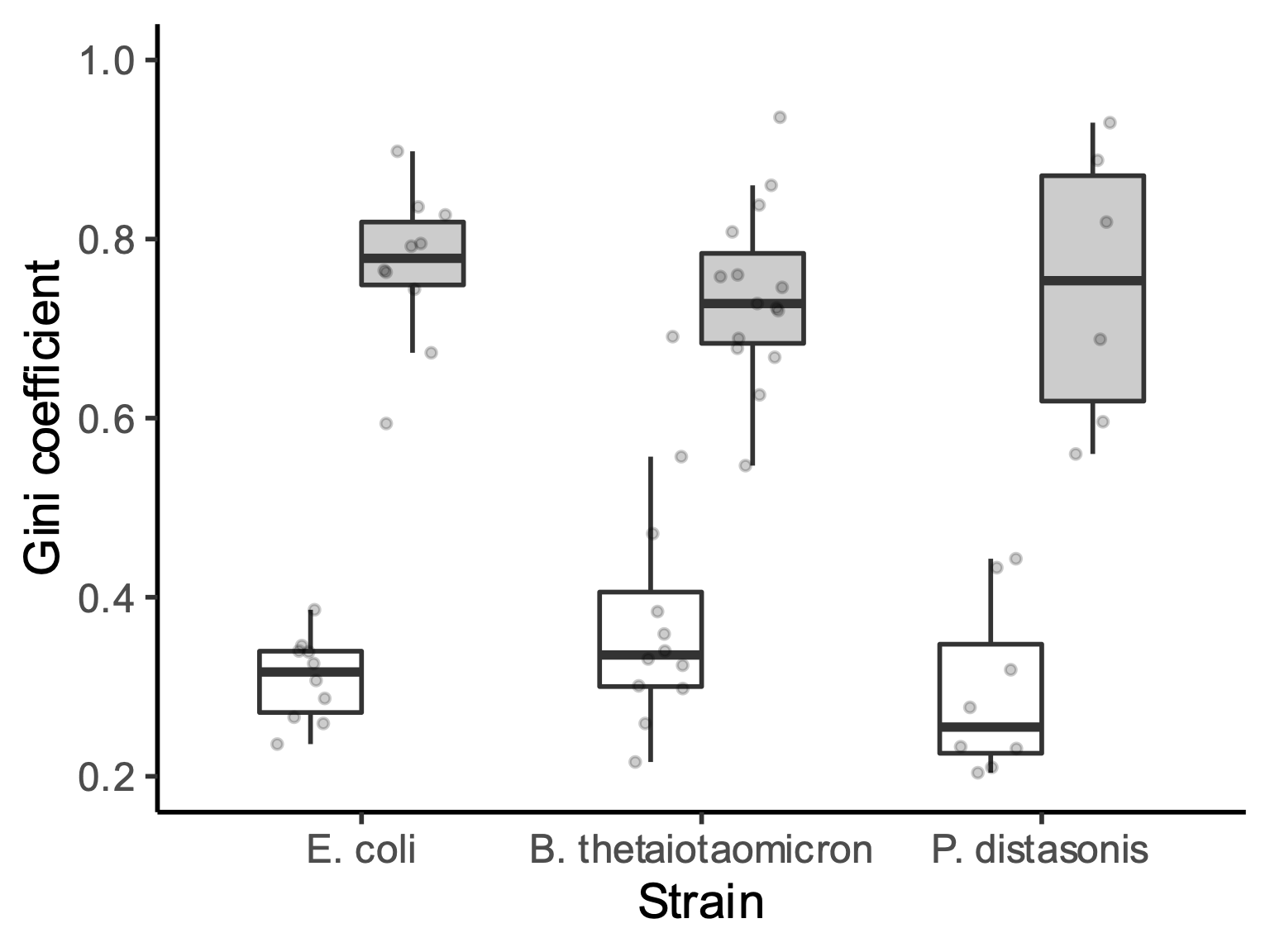


(10)

(10)

(12)

(15)

(8)

(6)

*Escherichia*

*coli*

*Bacteroides*

*thetaiotaomicron*

*Parabacteroides*

*distasonis*

**

**

**

**a**


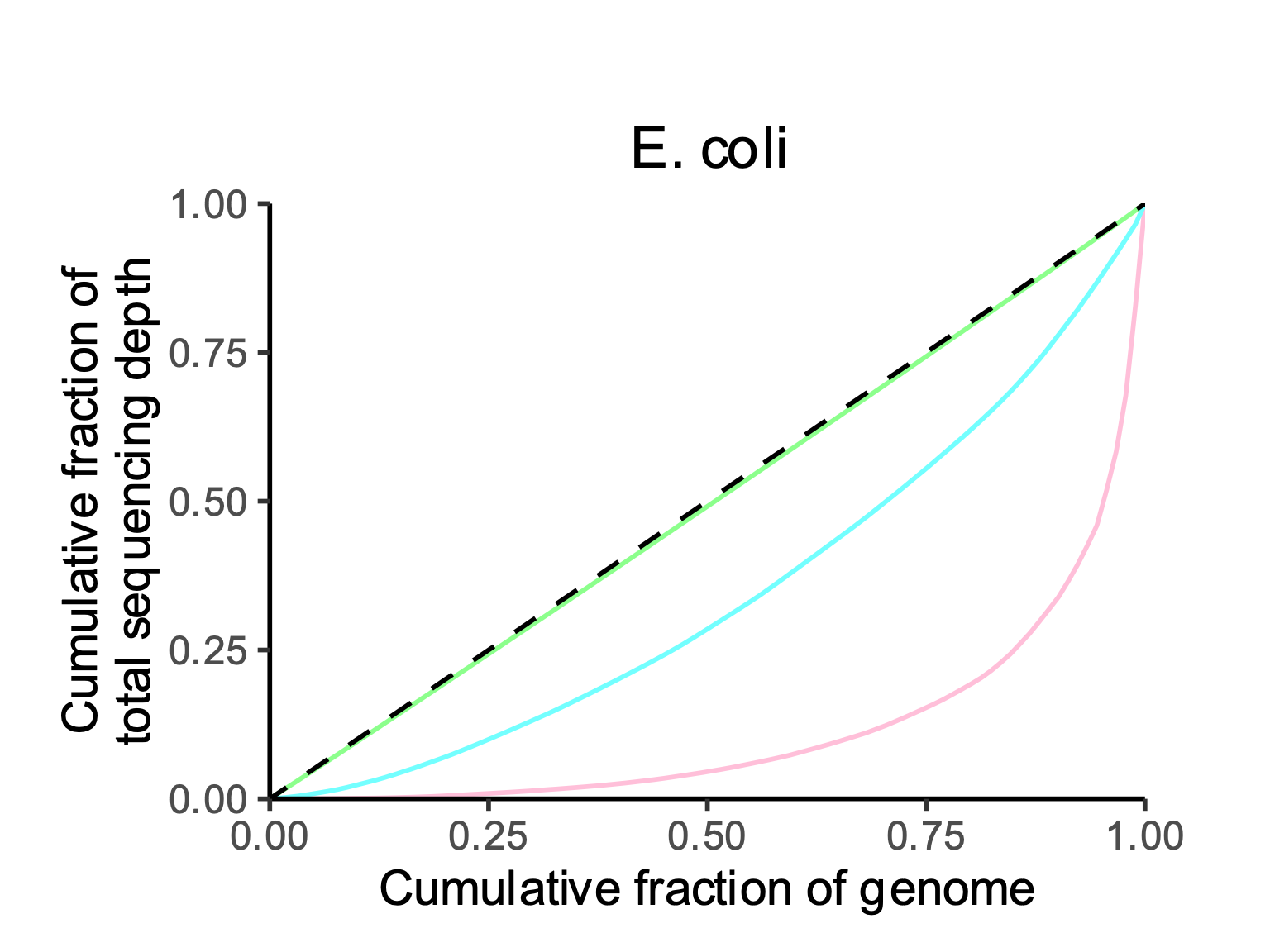

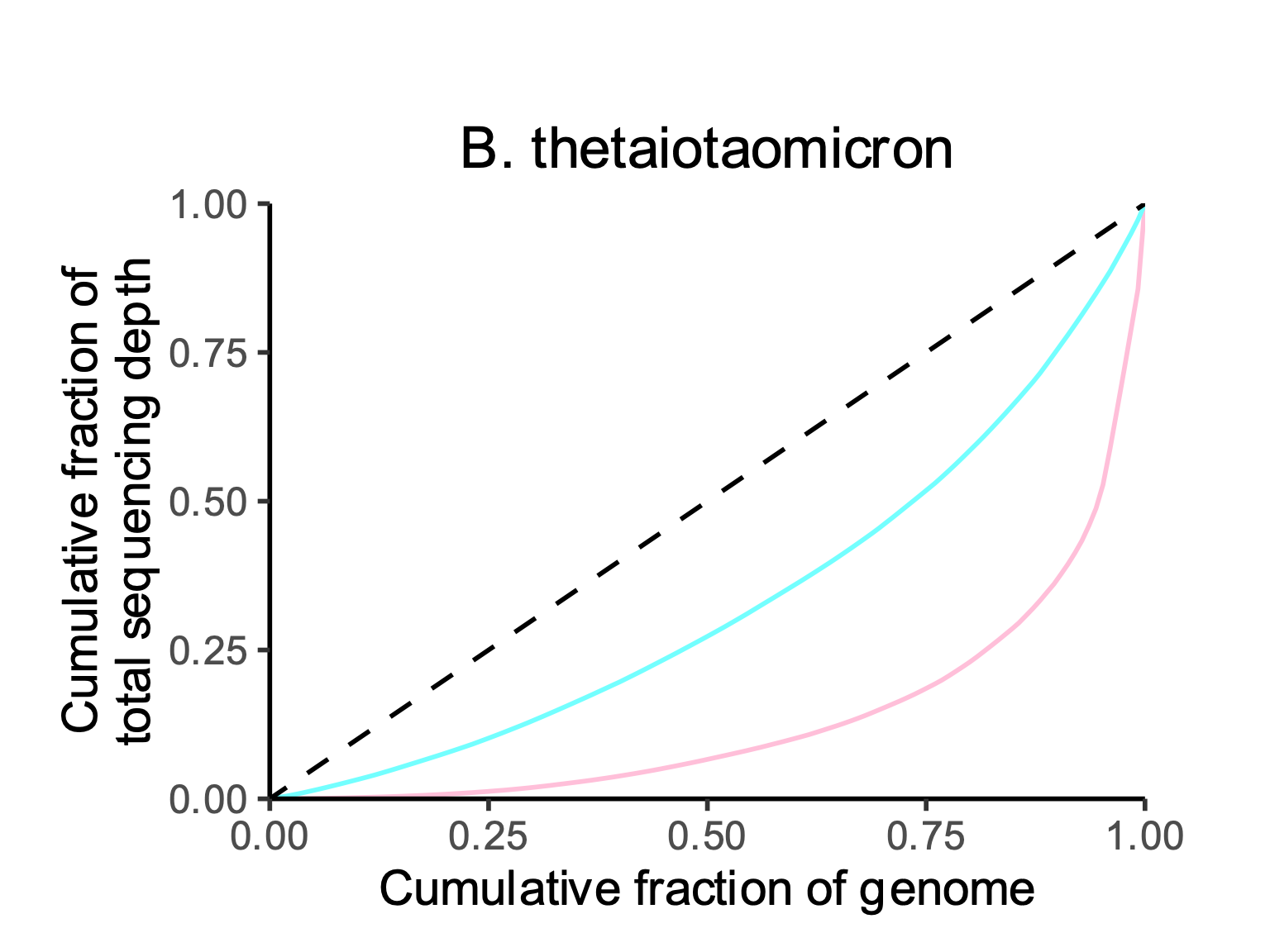

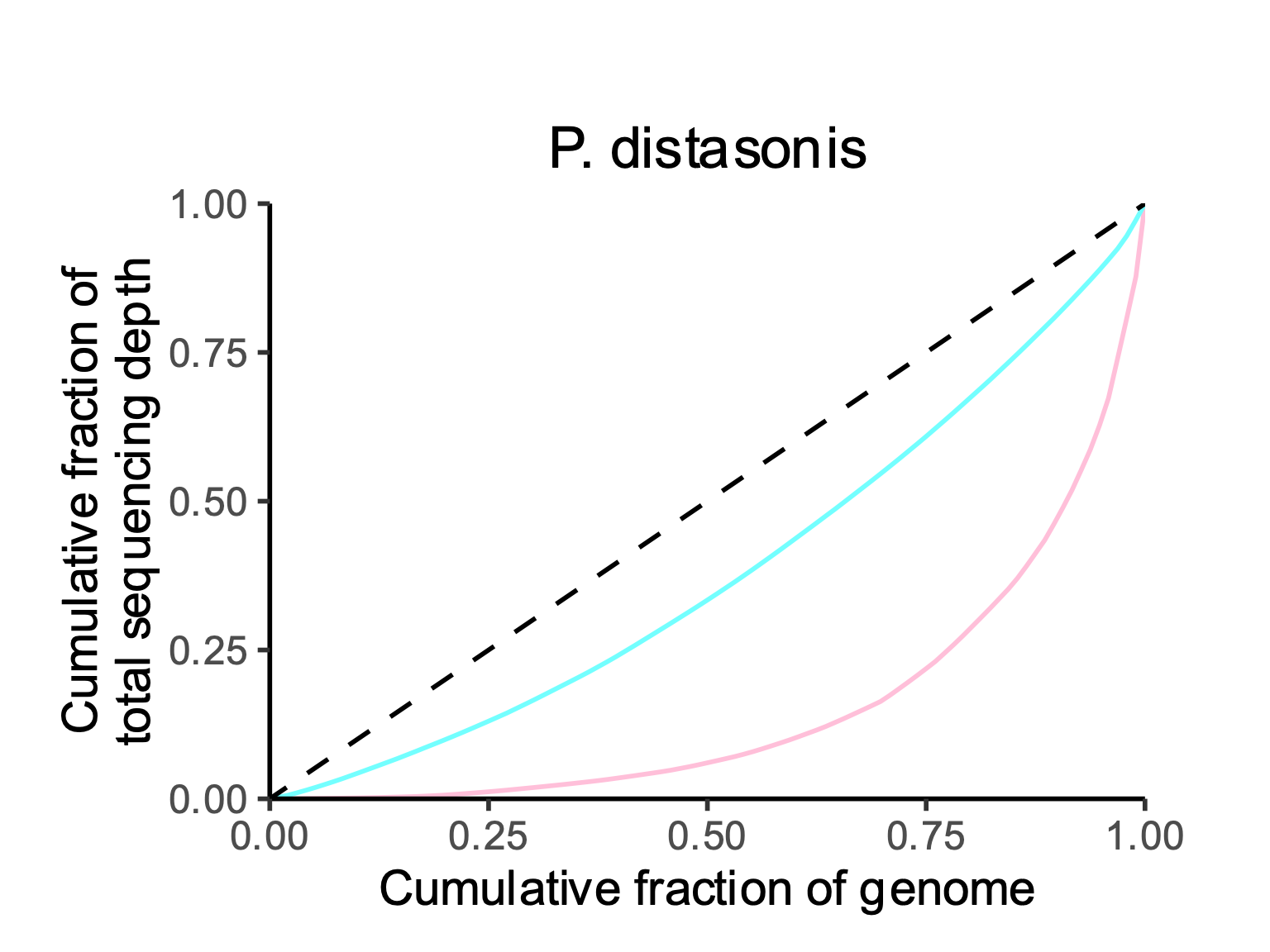


*Escherichia coli*

*Bacteroides thetaiotaomicron*

*Parabacteroides distasonis*

**b**

Fig. S6


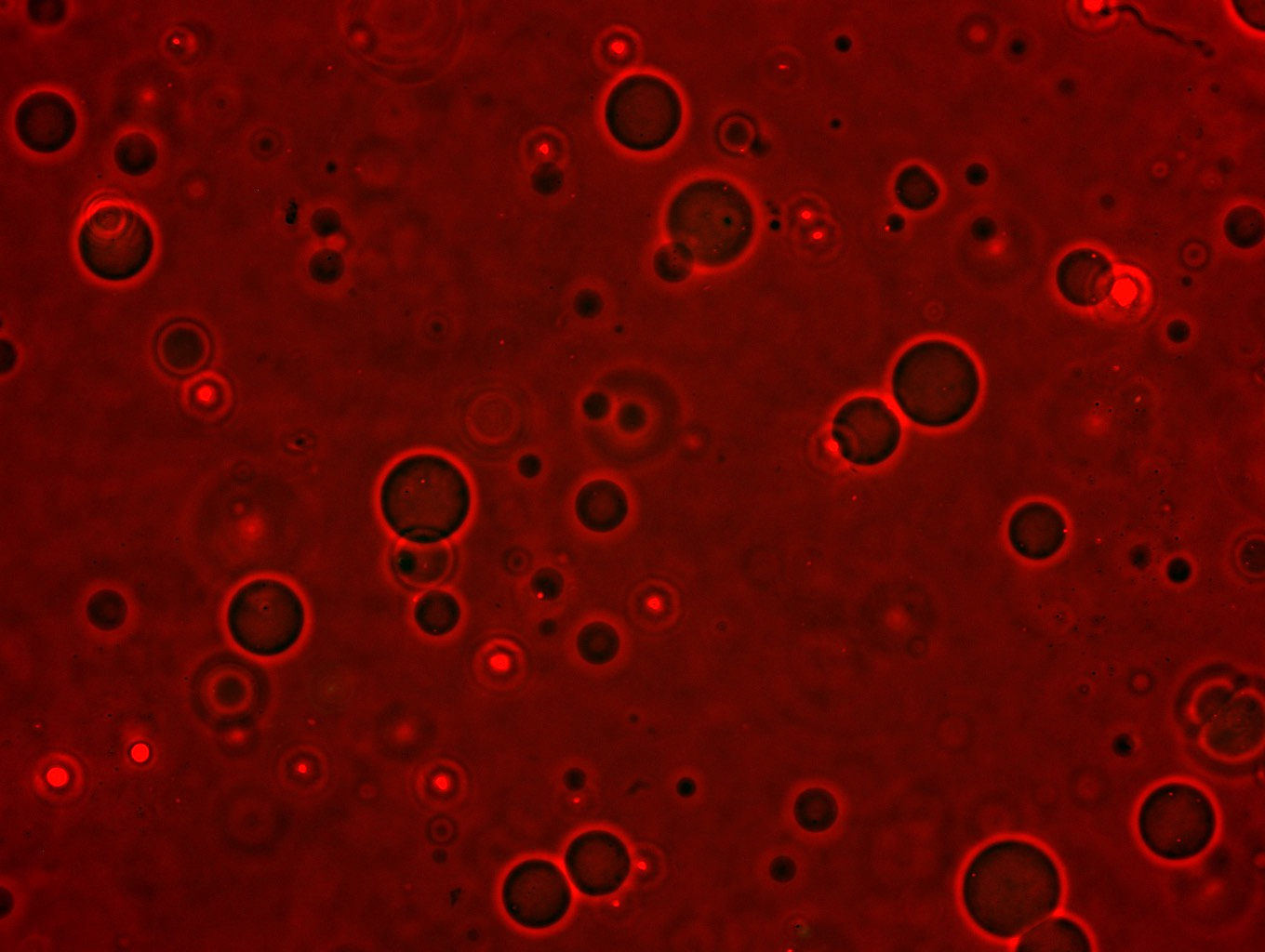

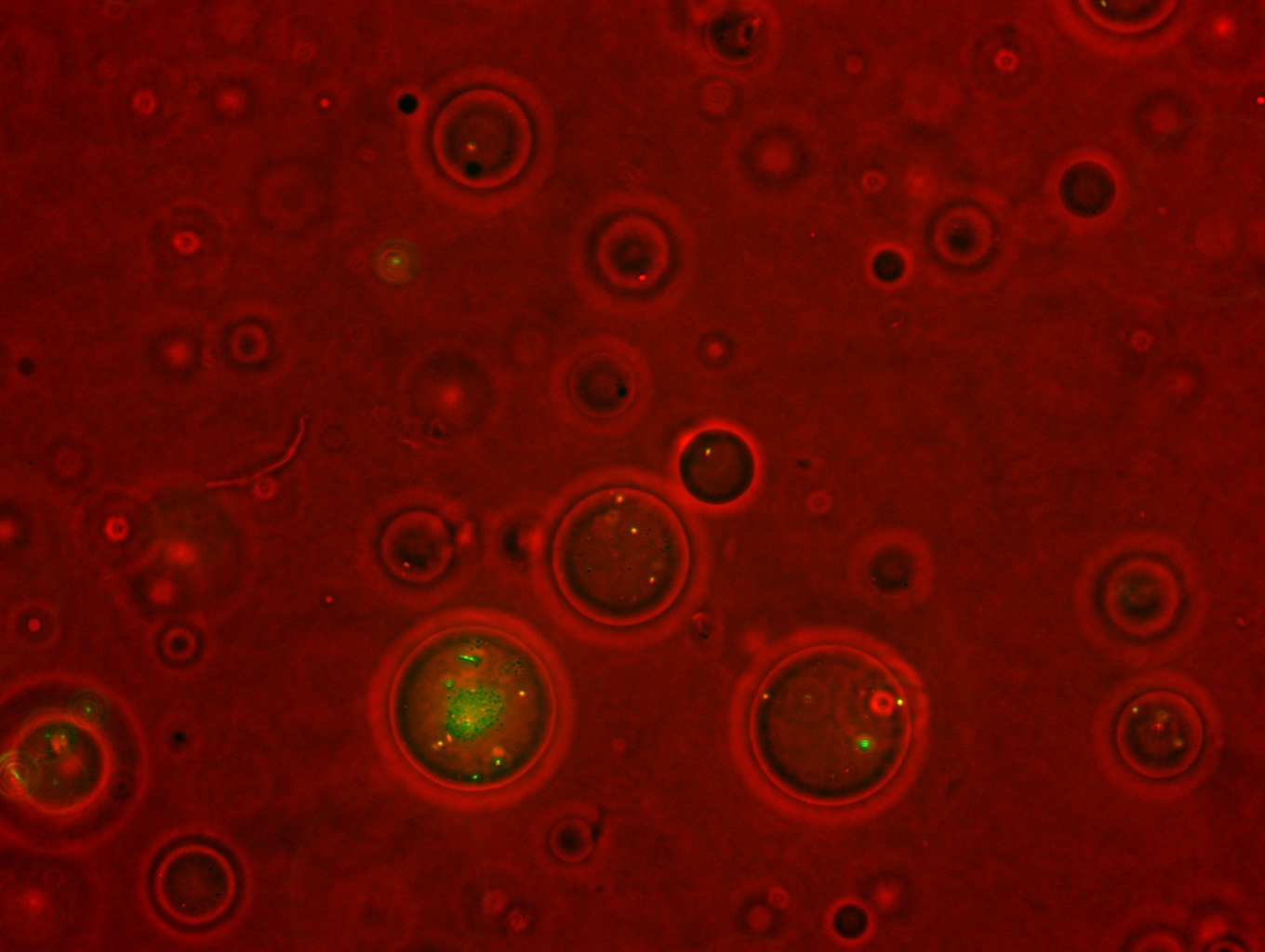


0

3.05×10^6^


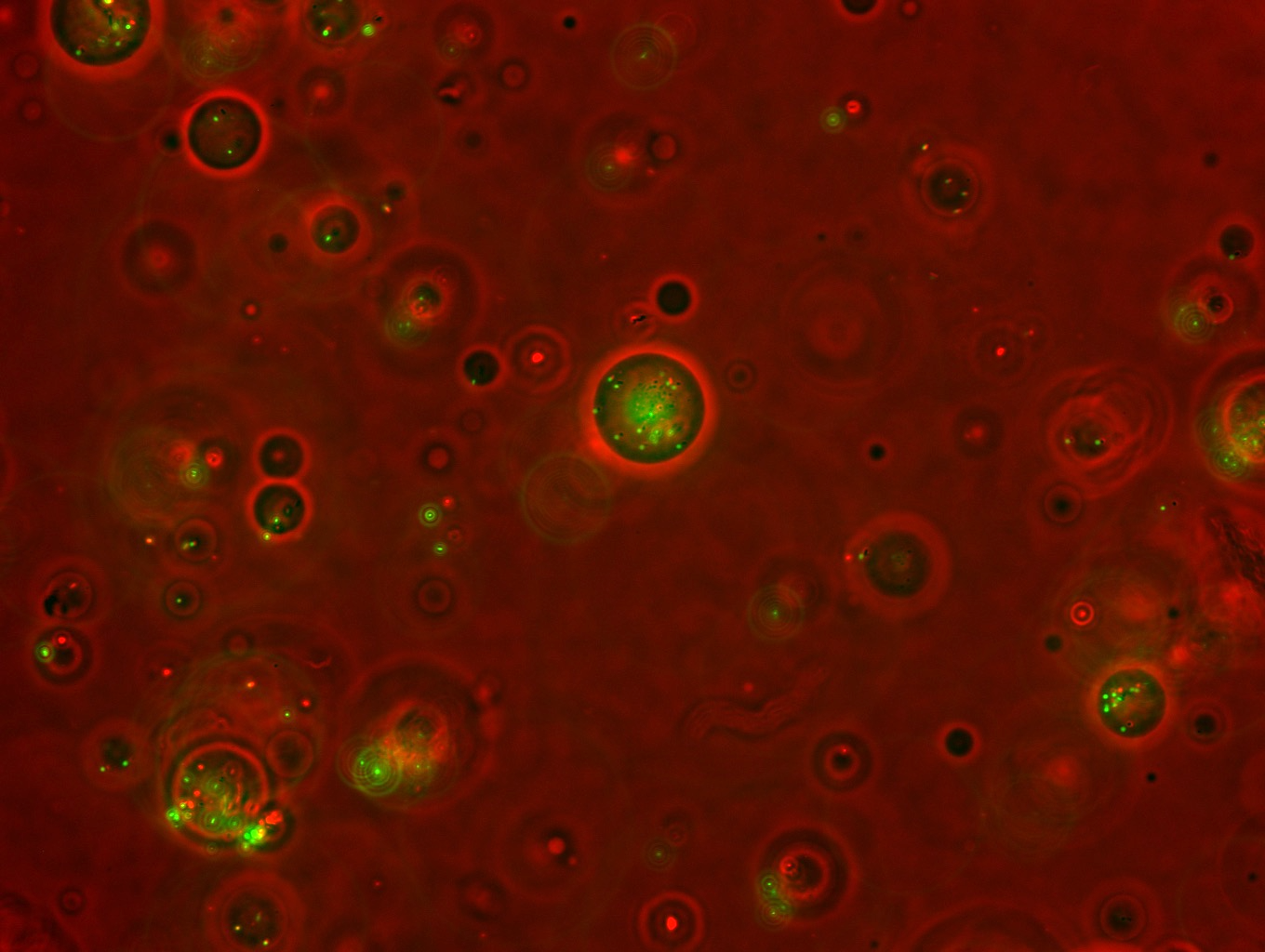

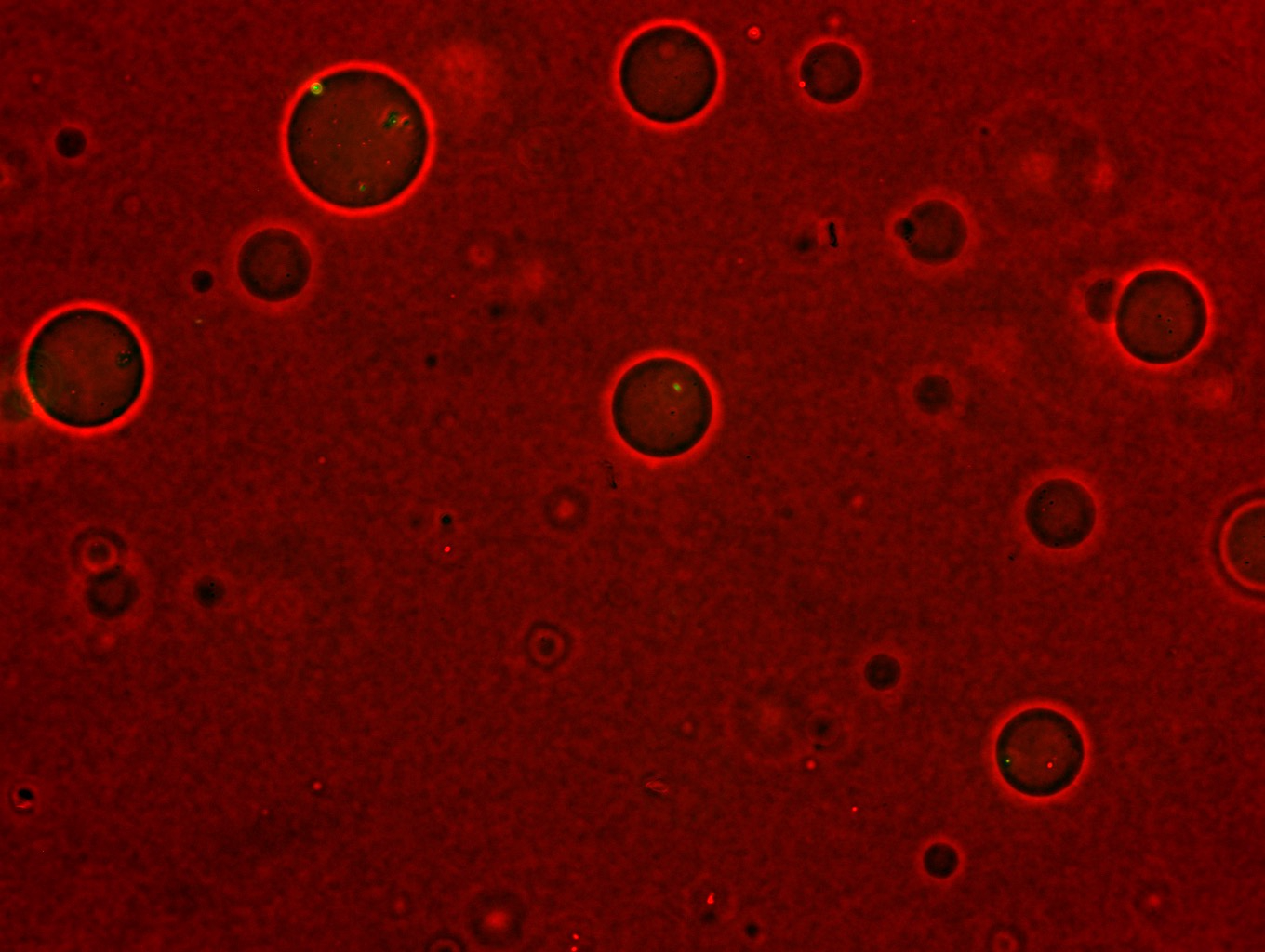


3.05×10^7^

3.05×10^8^

Fig. S7

500 µm
